# Cardiovascular risk algorithms in primary care: Results from the DETECT study

**DOI:** 10.1038/s41598-018-37092-7

**Published:** 2019-01-31

**Authors:** Tanja B. Grammer, Alexander Dressel, Ingrid Gergei, Marcus E. Kleber, Ulrich Laufs, Hubert Scharnagl, Uwe Nixdorff, Jens Klotsche, Lars Pieper, David Pittrow, Sigmund Silber, Hans-Ulrich Wittchen, Winfried März

**Affiliations:** 1 University of Heidelberg, Mannheim Medical Faculty, Mannheim Institute of Public Health, Social and Preventive Medicine, Mannheim, Germany; 2 University of Heidelberg, Mannheim Medical Faculty, Department of Internal Medicine V (Nephrology, Hypertensiology, Rheumatology, Endocrinology, Diabetology), Mannheim, Germany; 3 Clinic and Polyclinic of Cardiology, University Clinic Leipzig, Leipzig, Germany; 4 Medical University of Graz, Clinical Institute of Medical and Chemical Laboratory Diagnostics, Graz, Austria; 5 European Prevention Center, EPC GmbH, Düsseldorf, Germany; 6 German Research Center of Rheumatology Berlin, Leibnitz Institute, Berlin, Germany; 7 Charité Universitätsmedizin Berlin, Institute of Social Medicine, Epidemiology and Health Economics, Berlin, Germany; 8 Technical University Dresden, Medical Faculty, Institute of Clinical Pharmacology, Dresden, Germany; 9 Cardiology Outpatient Clinic Tal, Munich, Germany; 10 Technical University Dresden, Institute of Clinical Psychology and Psychotherapy, Dresden, Germany; 11 Max-Planck- Institute of Psychiatry, Munich, Germany; 12 Synlab Services GmbH, Synlab Academy, Mannheim, Augsburg Germany

## Abstract

Guidelines for prevention of cardiovascular diseases use risk scores to guide the intensity of treatment. A comparison of these scores in a German population has not been performed. We have evaluated the correlation, discrimination and calibration of ten commonly used risk equations in primary care in 4044 participants of the DETECT (Diabetes and Cardiovascular Risk Evaluation: Targets and Essential Data for Commitment of Treatment) study. The risk equations correlate well with each other. All risk equations have a similar discriminatory power. Absolute risks differ widely, in part due to the components of clinical endpoints predicted: The risk equations produced median risks between 8.4% and 2.0%. With three out of 10 risk scores calculated and observed risks well coincided. At a risk threshold of 10 percent in 10 years, the ACC/AHA atherosclerotic cardiovascular disease (ASCVD) equation has a sensitivity to identify future CVD events of approximately 80%, with the highest specificity (69%) and positive predictive value (17%) among all the equations. Due to the most precise calibration over a wide range of risks, the large age range covered and the combined endpoint including non-fatal and fatal events, the ASCVD equation provides valid risk prediction for primary prevention in Germany.

## Introduction

Cardiovascular diseases are the leading cause of death in Europe and worldwide^1^. In 2015, 17,7 million people died from cardiovascular diseases worldwide of which an estimated 7,4 million were due to coronary heart disease and 6,7 million due to stroke (www.who.int/mediacentre/factsheets/fs317/en/). In Germany, the direct costs for the treatment of cardiac and circulatory diseases are approximately 46 billion euros per year (2015) and they contribute to about one-sixth of the total healthcare costs, not including indirect costs of productivity losses (https://www.destatis.de/DE/ZahlenFakten/GesellschaftStaat/Gesundheit/Krankheitskosten/Krankheitskosten.html).

According to relevant international and national guidelines for cardiovascular disease prevention, the intensity of drug interventions in primary prevention depends on the assessment of an individual´s cardiovascular risk. To determine this risk, US American^2,3^, European^4,5^ and German^6–10^ guidelines recommend different risk equations.

The guideline of the NCEP/ATP (National Cholesterol Education Program Adult Treatment Panel) III from 2002^11^ and 2004^3^, the guidelines of the European Society of Cardiology (ESC) and the European Atherosclerosis Society (EAS) for prevention and for the treatment of dyslipidemia^4,5^ have recommended the Framingham risk score (FRS)^12^ and the ESC Heart Score (ESC-HS)^13^, respectively. Unlike other calculators, the ESC-HS provides the risk of cardiovascular mortality only, but not of non-fatal events and a factor for the conversion of the results of this algorithm into other is not available. The current North American guideline uses the ASCVD (atherosclerotic cardiovascular disease score, sometimes coined Pooled Cohort Equation) to account for the historical nature and ethnic population limitations of the FRS. The ASCVD specifically incorporates cohorts with individuals of Hispanic and African-American descent to allow its use in a contemporary US population^2^.

Besides these risk scores, the PROCAM (Prospective Cardiovascular Muenster Study) algorithm (www.chd-taskforce.com; www.assmann-stiftung.de) and the ARRIBA – algorithm (which is derived from the FRS, but has not previously been validated empirically) are commonly used in Germany.

The QRISK score^14^ and the ASSIGN score^15^, which have been derived from large United Kingdom primary care population datasets and which also include specific items such as deprivation indices and multiple ethnic subgroups, and the JBS3 risk calculator^16^ are common in the United Kingdom. The CUORE risk equation has been developed for Italy^17,18^. The UKPDS risk engine^19^ has been recommended for patients with diabetes mellitus only, but the NICE clinical guideline (CG181)^20^ has recommended QRISK for risk assessment in type 2 diabetes mellitus, because the UKPDS risk engine had significant bias. Since these risk scores are difficult to translate to other countries, we did not evaluate them in the current analysis.

The diversity of the remaining risk equations has prompted us to compare the discriminatory power and calibration of the main national and international risk calculators in the DETECT study (Diabetes and Cardiovascular Risk Evaluation: Targets and Essential Data for Commitment of Treatment) which included a representative German primary care population.

## Results

We studied 4044 patients, whose data were valid and fully available at the beginning of the study. The demographic and clinical characteristics of the study population are shown in Table 1. The mean age of the study population was 53.8 ± 13.7 years (18 to 93 years) at baseline, 65.3% were women. The prevalence rate of hypertension was 37.1%. Patients with diabetes mellitus were excluded. There were no major differences between the original third layer laboratory sample (n = 7519) of the DETECT study (for explanation see Methods) and the current study population (n = 4044, Supplementary Tables 1 and 2), and there were also no differences between participants living in East and West Germany (Supplementary Table 3).

**Table 1:** Characteristics of the study population.

Variable	Total	Female	Male	P^b^
Number	4044	2641	1403	
Age (years)	53.8 ± 13.7	53.5 ± 14.1	54.5 ± 13.1	0.034
HDL cholesterol (mg/dl)	57.2 ± 18.8	61.9 ± 18.6	48.4 ± 15.6	<0.0001
LDL cholesterol (mg/dl)	129.3 ± 33.3	128 ± 33.8	131.8 ± 32.4	0.0006
Cholesterol (mg/dl)	225.9 ± 41	226.1 ± 41.2	225.8 ± 40.5	0.8229
Triglycerides (mg/dl)^a^	118.7 (68.5,205.8)	107.8 (65.2,178.1)	142.4 (79,256.7)	<0.0001
HbA1c (%)	5.3 ± 0.3	5.3 ± 0.3	5.3 ± 0.3	0.0003
Glucose (mg/dl)	101.7 ± 16.3	99.9 ± 15.4	105 ± 17.4	<0.0001
hsCRP (mg/l)^a^	2 (0.7,6.4)	2.1 (0.7,6.7)	1.9 (0.6,5.7)	0.0002
Arterial hypertension, n (%)	1502 (37.1)	933 (35.3)	569 (40.6)	0.0012
Systolic blood pressure (mm Hg)	129.5 ± 17.5	128 ± 18.1	132.3 ± 15.8	<0.0001
Diastolic blood pressure (mm Hg)	79.6 ± 9.8	78.9 ± 1	81 ± 9.2	<0.0001
Smoking never/ex/current, n (%)	2291/819/934 (56.7/20.3/23.1)	1688/365/588 (63.9/13.8/22.3)	603/454/346 (43/32.4/24.7)	<0.0001
Positive family history, n (%)	1082 (26.8)	743 (28.1)	339 (24.2)	0.0072
BMI (kg/m²)	26.4 ± 4.6	26 ± 4.8	27.2 ± 4.1	<0.0001
**Medication**				
Antihypertensive medication, n (%)	1086 (26.9)	689 (26.1)	397 (28.3)	0.1361
Lipid-lowering medication, n (%)	313 (7.7)	176 (6.7)	137 (9.8)	0.0005
Antithrombotics, n (%)	226 (5.6)	121 (4.6)	105 (7.5)	0.0002
**Clinical endpoints during follow-up**				
Death, n (%)	75 (1.9)	39 (1.5)	36 (2.6)	0.0194
Cardiovascular death, n (%)	57 (1.4)	30 (1.1)	27 (1.9)	0.0495
Non-letal myocardial infarctions, n (%)	19 (0.5)	6 (0.2)	13 (0.9)	0.0031
PTCA/ACVB, n (%)	36 (0.9)	11 (0.4)	25 (1.8)	<0.0001
Stroke, TIA, n (%)	69 (1.7)	33 (1.3)	36 (2.6)	0.0031
Symptomatic peripheral arterial disease, n (%)	32 (0.8)	15 (0.6)	17 (1.2)	0.0386
Endpoint 1 (PROCAM-I, FRS-hard-CVE), n (%)	72 (1.8)	28 (1.1)	44 (3.1)	<0.0001
Endpoint 2 (Reynolds, ASCVD, ARRIBA), n (%)	101 (2.5)	43 (1.6)	58 (4.1)	<0.0001
Endpoint 3 (FRS-CHD1, FRS-CHD1), n (%)	132 (3.3)	58 (2.2)	74 (5.3)	<0.0001
Endpoint 4 (FRS-CVD), n (%)	220 (5.4)	106 (4)	114 (8.1)	<0.0001

^a^Values for triglycerides and hsCRP in parentheses correspond to logarithmic standard intervals.

^b^Student’s T test and exact Fisher’s test in the case of continuous and categorial variables, respectively.

**Figure 1:**
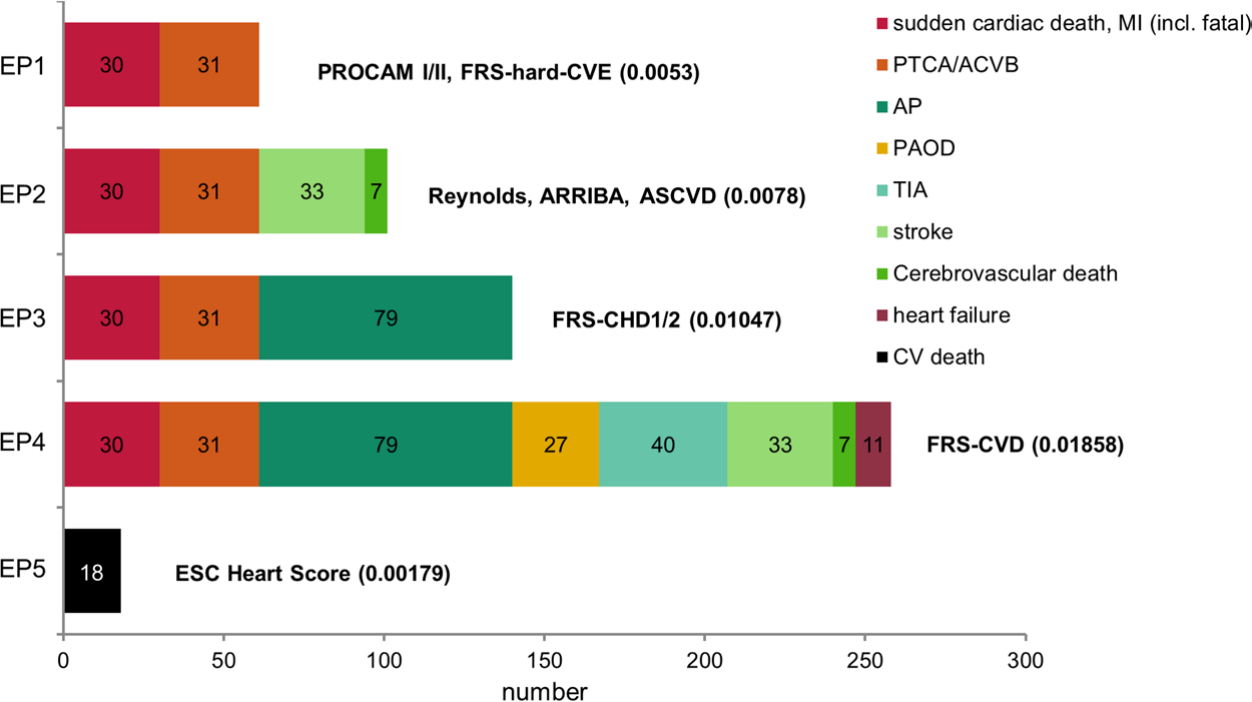
Absolute number of events for the end points of the examined risk scores in the DETECT study (n = 4044).

The characteristics of the risk algorithms compared in the DETECT study is shown in Table 2. The absolute number of endpoints and the event rates for each of the risk algorithms is shown in Fig. 1.

**Table 2:** Characteristics of risk algorithms.

	PROCAM-I	PROCAM-II	Reynolds	FRS-CVE	FRS-CHD1	FRS-CHD2	FRS-CVD	ARRIBA	ASCVD	ESC-HS
Data source/population	Employed men, Westfalen, Germany n = 5389	Employed men and women, Westfalen, Germany, n = 8130	Primary care population, USA F: Womens’ Health Study, n = 16.400; M: Physicians Health Study II, n = 10.724	Primary care population, Framingham, MA, USA; n = not provided	Primary care population, Framingham, MA, USA, n = 5345	Primary care population, Framingham, MA, USA, n = 5345	Primary care population, Framingham, MA, USA n = 8491	Primary care population, Framingham, MA, USA, not provided	Nonwhite and white (NHLBI-financed cohort studies) USA, n = 24.626	CHD-mortality,12 European studies n = 205.178
Age (years)	35–65	20–75	F: ≥ 45 M: 50–79	20–79	30–74	30–74	30–74	20–79	40–79	40–65
**Covariables**										
Gender	+*	+	+	+	+	+	+	+	+	+
Age	+	+	+	+	+	+	+	+	+	+
Family history (y/n)	+	+	+	−	−	−	−	−	−	−
Smoking (y/n)	+	+	+	+	+	+	+	+	+	+
Systolic blood pressure	+	+	+	+	+	+	+	+	+	+
Diastolic blood pressure	−	−	−	−	+	+	−	−	−	−
Antihypertensives (y/n)	−	−	−	+	−	−	+	+	+	−
Cholesterol	−	−	+	+	+	−	+	+	+	+
HDL cholesterol	+	+	+	+	+	+	+	+	+	+
LDL cholesterol	+	+	−	−	−	+	−	−	-	−
Triglycerides	+	+	−	−	−	−	−	−	−	−
hsCRP	−	−	+	−	−	-	−	−	−	−
Glucose	−	+	−	−	−	−	−	−	−	−
HbA1c	−	−	+**	−	−	−	−	−	−	−
Diabetes mellitus (y/n)	+	+	+**	−	+	+	+	+	+	−
Clinical endpoint	Sudden cardiac death, myocardial infarction (incl. fatal)	Sudden cardiac death, myocardial infarction (incl. fatal)	myocardial infarction, stroke, coronary revascularisation, cardiovascular death	Coronary death, myocardial infarction	Coronary death, myocardial infarction, coronary insufficiency, angina pectoris	Coronary death, myocardial infarction, coronary insufficiency, angina pectoris	Coronary death, myocardial infarction, coronary insufficiency, angina pectoris, heart failure, TIA, peripheral arterial obstructive disease, stroke (ischemic and hemorrhagic), cerebrovascular death	Coronary death, myocardial infarction, stroke (incl. fatal)	Coronary death, myocardial infarction, stroke (incl. fatal)	cardiovascular death (ICD-9 codes 401–414, 426–443) with exception of ICD-9 426.7,429.0, 430.0, 432.1, 437.3, 437.4, 437.5 and 798.2
Endpoint (EP) for validation in DETECT	1 Sudden cardiac death, myocardial infarction (incl. fatal), coronary bypass, PTCA	1 Sudden cardiac death, myocardial infarction (incl. fatal), coronary bypass, PTCA	2 Sudden cardiac death, myocardial infarction (incl. fatal), coronary bypass, PTCA, stroke, cerebrovascular death	1 Sudden cardiac death, myocardial infarction (incl. fatal), coronary bypass, PTCA	3 Sudden cardiac death, myocardial infarction (incl. fatal), coronary bypass, PTCA, angina pectoris	3 Sudden cardiac death, myocardial infarction (incl. fatal), coronary bypass, PTCA, angina pectoris	4 Sudden cardiac death, myocardial infarction (incl. fatal), coronary bypass, PTCA, angina pectoris TIA, peripheral arterial occlusive disease, stroke, cerebrovascular death, heart failure (NYHA III-IV)	2 Sudden cardiac death, myocardial infarction (incl. fatal), coronary bypass, PTCA, stroke, cerebrovascular death	2 Sudden cardiac death, myocardial infarction (incl. fatal), coronary bypass, PTCA, stroke, cerebrovascular death	5 sudden cardiac death, fatal MI, fatal stroke

^*^Recommended risk score for women = 0.25*(risk score for men), **only for women.

### Correlations

Supplementary Table 4 shows parametric (Pearson) and nonparametric (Spearman) correlation coefficients of 10-years risks in 2463 study participants calculated with the data of the first survey (subset of the sample between 40 and 65 years of age as described in “Methods”). All algorithms correlate well with each other. The Pearson and Spearman correlation coefficients are similar. The FRS-CVD has the best correlations with all other algorithms. Supplementary Fig. 1 shows scatter diagrams in which results of the FRS-CVD are placed on the x-axis and the other algorithms on the y-axis. The relative “closeness” of the algorithms to each other is shown graphically in Supplementary Fig. 2. FRS-CHD1, FRS-CHD2, FRS-CVD and ASCVD are closest to each other, respectively.

### Discrimination

For all algorithms, we calculated AUCs and Harrel C statistics for the clinical endpoint belonging to the respective algorithm and for the other endpoints (Table 3). For broad clinical endpoints (EP3 and EP4; for definitions see Methods) the discriminatory power of all algorithms was lower than for narrowly defined endpoints (EP1 and EP2). This finding did not depend on whether an algorithm was originally developed for a wide or narrow endpoint. For example: the AUC and Harrell C statistics of the FRS-CVD for EP4 (associated endpoint) are 0.72 and 0.72, respectively; AUC and Harrell C statistics of the FRS-CVD for EP1 (endpoint PROCAM or FRS hardCVE) are 0.78 and 0.80, respectively.

**Table 3:** AUCs and Harrell’s C-statistics of risk algorithms related to four composite end points (n = 2463, in brackets: 95%-confidence intervals).

Endpoint	2 Reynolds, ASCVD, ARRIBA		1 PROCAM-I, FRS-hard-CVE		3 FRS-CHD1, FRS-CHD2		4 FRS-CVD		AUCs and Harrell’s Cstatistics from literature^11-13,34,38,49-52^	
Number of events (baseline age 40–65 years)	29		20		50		89			
Annual rate of events (baseline age 40–65 years)	0.00366		0.00252		0.00633		0.01137			
	AUC	Harrell-C	AUC	Harrell-C	AUC	Harrell-C	AUC	Harrell-C	AUC	Harrell-C
Reynolds	**0.79 (0.73–0.86)**	**0.80 (0.74–0.86)**	0.80 (0.70–0.89)	0.82 (0.75–0.89)	0.73 (0.66–0.79)	0.72 (0.65–0.78)	0.71 (0.66–0.76)	0.71 (0.66–0.76)	Not provided	0.7 (CHD, m), 0.71 (CVD, f)
ASCVD	**0.76 (0.68–0.84)**	**0.77 (0.70–0.84)**	0.78 (0.67–0.88)	0.80 (0.71–0.88)	0.73 (0.66–0.79)	0.72 (0.65–0.79)	0.72 (0.67–0.77)	0.71 (0.66–0.77)	0.71–0.82	Not provided
PROCAM-I	0.76 (0.70–0.82)	0.77 (0.72–0.83)	**0.79 (0.71–0.88)**	**0.81 (0.74–0.88)**	0.70 (0.64–0.77)	0.70 (0.63–0.77)	0.70 (0.64–0.75)	0.69 (0.63–0.74)	0.83 (f)	Not provided
FRS-CHD1	0.75 (0.67–0.83)	0.75 (0.67–0.83)	0.73 (0.61–0.84)	0.75 (0.65–0.86)	**0.68 (0.62–0.75)**	**0.69 (0.63–0.76)**	0.68 (0.63–0.73)	0.68 (0.63–0.74)	0.74 (m), 0.77 (f)	Not provided
FRS-CHD2	0.74 (0.66–0.82)	0.75 (0.67–0.83)	0.75 (0.64–0.86)	0.78 (0.68–0.87)	**0.68 (0.61–0.75)**	**0.69 (0.62–0.76)**	0.68 (0.63–0.73)	0.68 (0.63–0.74)	0.73 (m), 0.77 (f)	Not provided
FRS-CVD	0.77 (0.7–0.84)	0.78 (0.71–0.85)	0.78 (0.67–0.88)	0.80 (0.72–0.89)	0.73 (0.66–0.79)	0.74 (0.68–0.8)	**0.72 (0.67–0.76)**	**0.72 (0.66–0.77)**	Not provided	0.76 (m), 0.79 (f)
FRS-hard-CVE	0.75 (0.68–0.82)	0.75 (0.67–0.82)	**0.78 (0.68–0.88)**	**0.79 (0.71–0.88)**	0.72 (0.65–0.78)	0.71 (0.64–0.78)	0.70 (0.65–0.75)	0.69 (0.64–0.75)	Not provided	Not provided
ARRIBA	**0.77 (0.7–0.84)**	**0.77 (0.70–0.84)**	0.80 (0.71–0.9)	0.82 (0.73–0.9)	0.71 (0.64–0.78)	0.71 (0.64–0.78)	0.70 (0.65–0.75)	0.7 (0.65–0.75)	Not provided	Not provided
ESC	0.78 (0.71–0.85)	0.74 (0.65–0.82)	0.79 (0.69–0.89)	0.77 (0.65–0.89)	0.74 (0.68–0.8)	0.69 (0.61–0.76)	0.72 (0.67–0.76)	0.67 (0.62–0.72)	0.84 (0.79–0.88)	Not provided

bold: Harrell-C-Statistics for the endpoint belonging to the respective algorithm.

We also calculated continuous net reclassification improvements according to Pencina *et al*.^21^ using each of the scores once as a reference and then comparing it to all others (Supplementary Table 5). This revealed significant reclassification of individuals in 18 out of the 36 pairwise comparisons.

### Calibration

**Figure 2:**
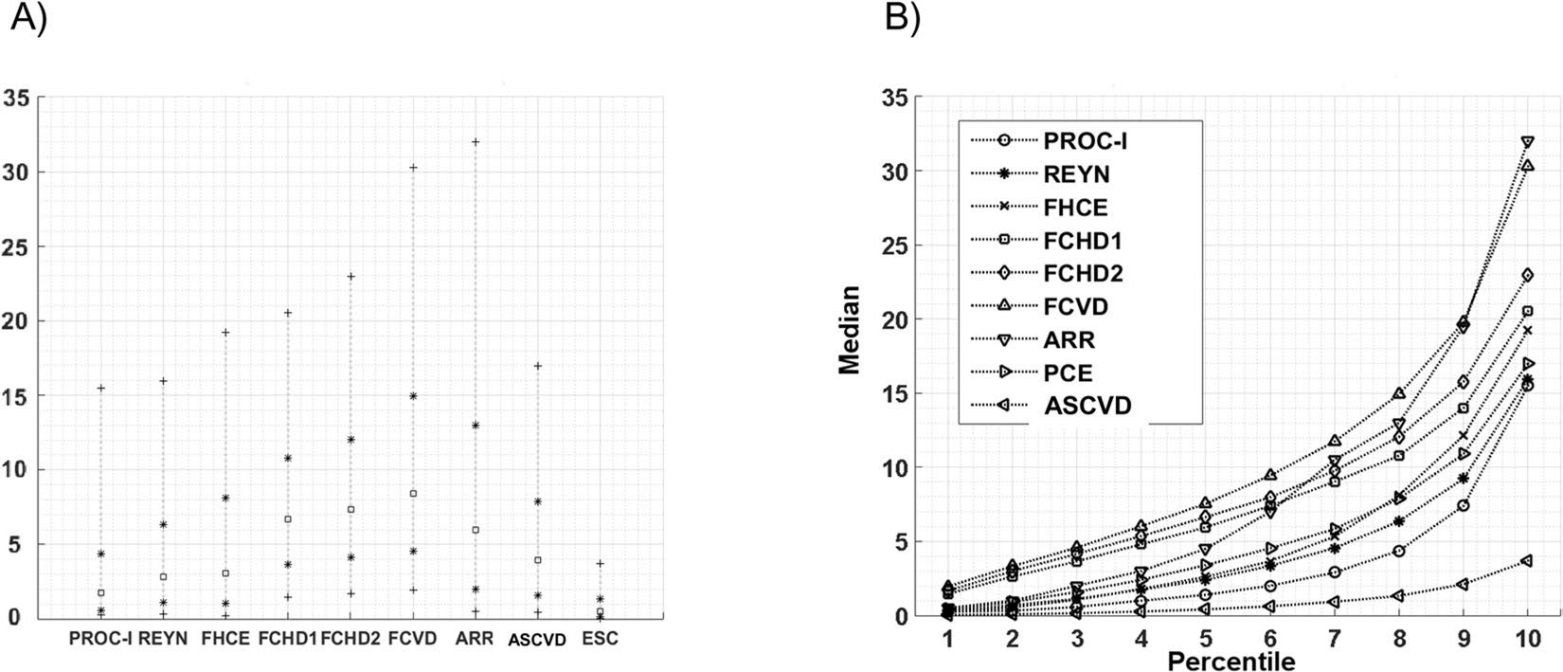
Distribution of results of the risk scores from 2463 participants of the DETECT study at baseline. (**A**) The distribution of calculated risks is illustrated by the fifth, 25th, 50th (median), 75th and 95th percentile. (**B**) The estimated risks were broken down into percentiles. Abscissa: percentile. Ordinate: median risk in each percentile.

Figure 2A shows the fifth, 25th, 50th (median), 75th and 95th percentiles of the risk equations. In terms of the medians, the FRS-CVD provides the highest absolute 10-year risk (8.4%), followed by FRS-CHD2 (7.3%), FRS-CHD1 (6.7%) and ARRIBA (6.0%). ASCVD (4.0%), FRS-hard CVE (3.1%), Reynolds (2.9%), PROCAM I (2.6%) and PROCAM II (2.0%) produced lower median risks, ESC-HS shows the lowest 10-year risk (0.5%). The results of the risk equations are shifted to the right side of the normal distribution. Differences exist mainly at high risk (Fig. 2B). ARRIBA has a particularly skewed distribution.

Assuming arbitrarily that 25% of the DETECT population were at high risk and eligible for intervention (exceeding the 75th percentile), the following thresholds for the calculated 10-year risk would be assigned: FRS-CVD (EP4) 14.9%; ARRIBA (EP2) 13.0%; FRS-CHD2 (EP3) 12.1%; FRS-CHD1 (EP3) 10.8%; FRS-hard CVE (EP1) 8.1%; ASCVD (EP2) 7.9%; PROCAM II (EP1) 7.0%; Reynolds (EP2) 6.4%; PROCAM I (EP1) 4.4% and ESC-HS (EP5) 1.3%.

Assuming again arbitrarily that only 10% of the DETECT population were at high risk and eligible for intervention (exceeding the 90th percentiles of risk), the following threshold values would result: FRS-CVD 23.5%, ARRIBA 18.9%, FRS-CHD2 18.9%, FRS-CHD1 16.7%, FRS-hard CVE 14.9%, ASCVD 12.9%, PROCAM II 14.5%, Reynolds 11.5%, PROCAM I 10.2% and ESC-HS 2.7%. Together, this demonstrates that the algorithms are calibrated differently, in part, but not exclusively, due to the broadness of the associated clinical endpoints. The calculated risks are not linearly convertible, they diverge differently at high and low risks.

**Figure 3:**
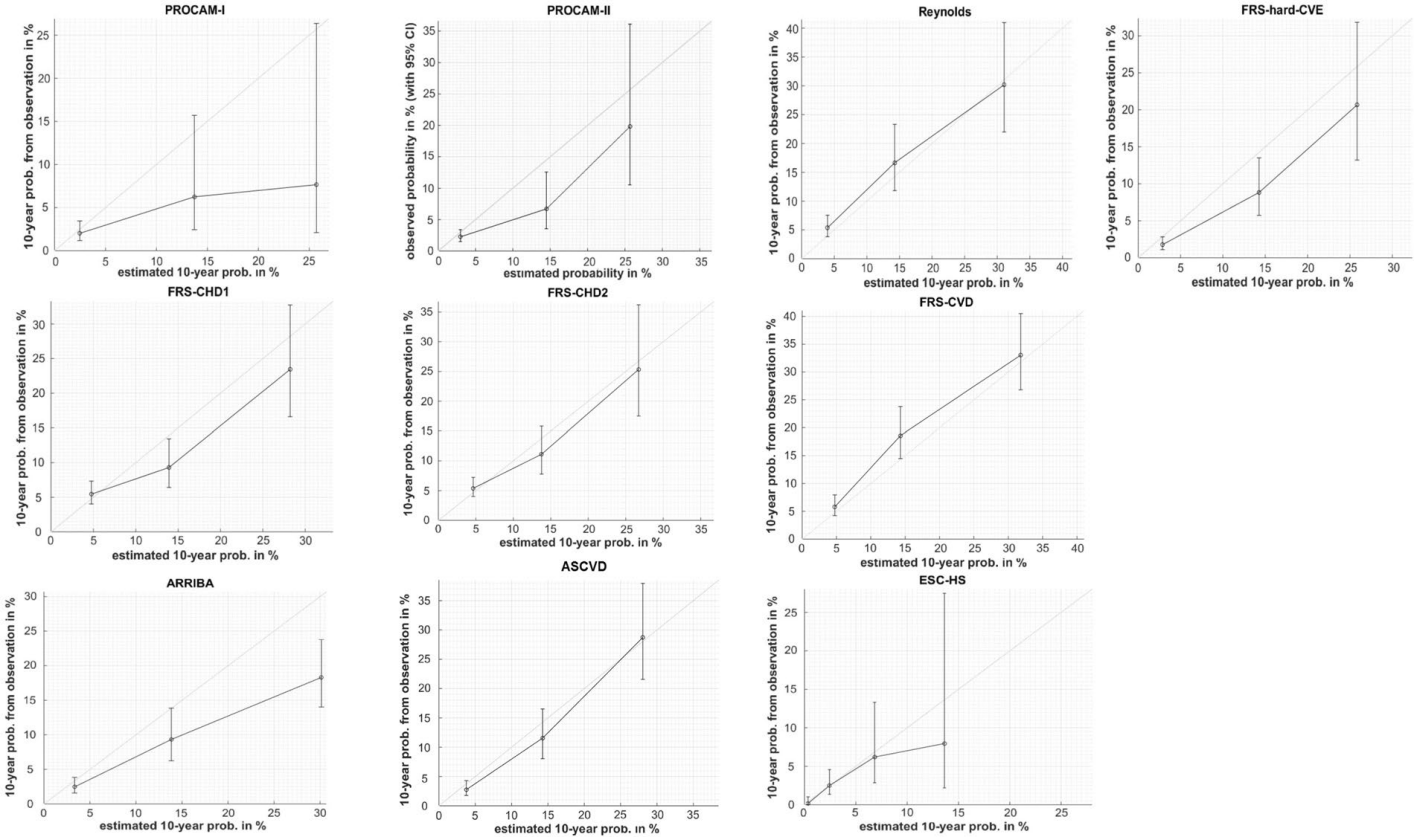
Calibration of risk scores. The estimated risks were divided in risk groups <10%, 10–20% and >=20%. In each of the resulting risk groups the average values (x-axis) were projected against the 10-year relative frequencies of corresponding endpoints (ordinate). The error bars represent 95% confidence intervals.

Figure 3 compares predicted and observed incidence rates. The incidence rates predicted by Reynolds, ASCVD and ESC-HS are consistent with the observed ones, whereby the results for the ESC-HS in the high-risk group have to be interpreted with caution because of the large confidence interval and the low mortality rate in this group. ARRIBA, PROCAM I, PROCAM II, FRS hard-CVE, FRS-CHD1 and FRS-CHD2 overestimate the actual risk in the middle- and/or high-risk group. FRS-CVD slightly underestimates the risk at medium risk.

In the category of the highest risk of the ESC-HS (more than 10 percent) not a single event occurred in the age group of 40 to 65 years. For this reason, the calibration of the ESC-HS could not be evaluated. When we extended the age range to 40–79 years, the ESC-HS was in good agreement with observed incidence rates for the risk groups 0–1%, 1–5% and 5–10%, while at higher risks (above 10 percent) the calculated risk still exceeded the observed one. Predicted and observed incidence rates were significantly different by the Hosmer-Lemeshow test (Table 4) for PROCAM I, FRS-CHD1, FRS-CVD, FRS-hard CVE and ARRIBA. ASCVD and Reynolds showed the best agreement between observed and predicted incidence rates. The PROCAM II score is not included in Table 4, because it provides only five discrete values which cannot be broken down into deciles. The p-value 0.94 for ESC-HS in the age group 40–79 years is to be considered with caution because of the low numbers of events.

**Table 4:** Hosmer-Lemeshow statistics.

Score	p-value
Reynolds	0.40
ASCVD	0.71
PROCAM-I	0.03
FRS-CHD1	0.04
FRS-CHD2	0.33
FRS-CVD	0.02
FRS-hard-CVE	0.03
ARRIBA	<0.01
ESC-HS	0.94^*^

*Age: 40–79 years (n = 3292).

### Thresholds for intervention

Table 5 compares the performance of all algorithms at the thresholds of 5, 10 and 20 percent 10-year risk (or 1, 2.5 and 5 percent, respectively, for the ESC-HS). This simulation has been conducted to delineate an optimum threshold of risk above which intervention and treatment should be considered.

**Table 5:** Comparison of methods for cardiovascular risk assessment in participants of the DETECT study at threshold levels 5, 10 und 20% risk for an event in 10 years (1, 2.5 and 5% for ESC-HS, respectively).

	Reynolds	ASCVD	PROCAM-I	PROCAM-II	FRS-CHD1	FRS-CHD2	FRS-CVD	FRS-hard-CVE	ARRIBA	ESC-HS
Number of individuals	2708	3292	2268	3783	3632	3632	3632	3928	3928	3292**
Threshold level	5	5	5	5	5	5	5	5	5	1
High risk*, n (%)	1523 (56.2)	1823 (55.4)	567 (25)	1160 (30.7)	2373 (65.3)	2211 (60.9)	2510 (69.1)	1581 (40.2)	2220 (56.5)	1579 (48)
Relative risk	7.69	11.28	4.13	3.05	5.31	5.14	6.62	10.18	7.48	19.53
Sensitivity	0.91	0.93	0.56	0.57	0.91	0.89	0.93	0.87	0.93	0.94
Specificity	0.48	0.48	0.76	0.70	0.37	0.42	0.35	0.62	0.47	0.53
PVP	0.17	0.14	0.06	0.07	0.11	0.12	0.19	0.09	0.12	0.03
PVN	0.98	0.99	0.98	0.98	0.98	0.98	0.97	0.99	0.98	1.00
Diagnostic efficiency	0.52	0.52	0.75	0.70	0.41	0.45	0.43	0.63	0.5	0.54
Threshold level	10	10	10	10	10	10	10	10	10	2.5
High risk*, n (%)	891 (32.9)	1156 (35.1)	257 (11.3)	528 (14)	1235 (34)	1068 (29.4)	1640 (45.2)	929 (23.7)	1544 (39.3)	879 (26.7)
Relative risk	4.21	6.9	3.61	3.49	2.63	3.00	4.50	7.22	6.01	14.64
Sensitivity	0.66	0.78	0.30	0.35	0.56	0.54	0.78	0.68	0.82	0.84
Specificity	0.71	0.69	0.89	0.87	0.68	0.73	0.60	0.78	0.64	0.74
PVP	0.22	0.18	0.07	0.09	0.13	0.15	0.24	0.12	0.14	0.05
PVN	0.95	0.97	0.98	0.97	0.95	0.95	0.94	0.98	0.98	1.00
Diagnostic efficiency	0.71	0.69	0.88	0.85	0.67	0.71	0.63	0.78	0.65	0.74
Threshold level	20	20	20	20	20	20	20	20	20	5
High risk*, n (%)	329 (12.1)	444 (13.5)	70 (3.1)	127 (3.4)	357 (9.8)	290 (8)	680 (18.7)	247 (6.3)	791 (20.1)	369 (11.2)
Relative risk	4.02	5.87	3.69	6.82	3.99	4.1	3.61	7.25	4.82	5.76
Sensitivity	0.34	0.47	0.09	0.18	0.28	0.25	0.44	0.31	0.56	0.42
Specificity	0.90	0.90	0.97	0.97	0.92	0.94	0.85	0.95	0.82	0.89
PVP	0.30	0.29	0.08	0.20	0.23	0.25	0.33	0.21	0.19	0.07
PVN	0.92	0.95	0.98	0.97	0.94	0.93	0.90	0.97	0.96	0.99
Diagnostic efficiency	0.84	0.86	0.95	0.94	0.87	0.88	0.79	0.92	0.8	0.89

*Risk ≥ threshold value in 10 years **Age: 40–79 years.

Relative risk: Observed relative risk of high-risk group compared to the low-risk group.

Sensitivity: Proportion of people with a calculated risk ≥ threshold in 10 years related to all persons, in which the cardiovascular event occurs for the corresponding score.

Specificity: proportion of individuals with a calculated risk < threshold in 10 years related to all persons without a cardiovascular event for the corresponding score.

PVP (Predictive value of the positive tests): proportion of people with a cardiovascular event belonging to the calculated score to all people with a calculated risk ≥ threshold.

PVN (Predictive value of the negative tests): proportion of people without a cardiovascular event belonging to the calculated score to all people with a calculated risk < threshold.

Diagnostic efficiency: the ratio of correct-predicted and correctly excluded cardiovascular events in the total cohort.

At the threshold of 5% (1% threshold for ESC-HS) the sensitivity of all algorithms for the corresponding endpoints is high; it varies between 56% (PROCAM) and 93% (FRS-CVD and ASCVD) or 94% (ESC-HS). At the 5% threshold, between 25 (PROCAM) and 69 (FRS-CVD) percent of the population would qualify for an intervention. The relationship between sensitivity and number of treated persons appears most favorable for the ASCVD; with a sensitivity of 93%, placing 55% of the population in need of treatment. The ESC-HS provides similar results, with a sensitivity of 94%, and 48% of the population in need of treatment. If the calculated risk was under 5%, the predictive value of negative tests for all algorithms is 99% or greater, saying that falling below this threshold an event occurring within the next 10 years is very unlikely.

This also applies to a risk threshold of 10% (threshold of 2.5% for the ESC-HS). The sensitivity varies between 30% (PROCAM-I) and 82% (ARRIBA) or 84% (ESC-HS); when using the ARRIBA almost 40% of the population would still be treated. ASCVD and FRS-CVD (78%) reveal slightly lower sensitivities. With FRS-CVD, 45% of the population would be treated, when using the ASCVD 35% of individuals would require intervention. Using ESC-HS only 27% would qualify for treatment. The relationship between sensitivity and the population that needs intervention appears optimal for the ASCVD (endpoint: non-fatal and fatal events).

The predictive value of a negative tests remains high even at the risk threshold of 20% (5% threshold for the ESC-HS). At risks above 20%, the sensitivity of the algorithms is between 9% (PROCAM-I) and 56% (ARRIBA). Slightly lower sensitivity than ARRIBA have the ASCVD (47%), the FRS-CVD (44%) and the ESC-HS (42%). Among the four algorithms with high sensitivity the ASCVD and the ESC-HS have the highest specificity (90% and 89%).

## Discussion

While large-scale validations of risk scores already exist in US populations^22–27^, this is the first comprehensive analysis of major cardiovascular risk algorithms in a German primary care setting. Beyond the scores currently recommended by international guidelines (ASCVD, ESC-HS), we also included earlier Framingham scores because they had been used to generate the ARRIBA score which is widely used by general practitioners in Germany and the PROCAM score which has been developed in Germany. Basically, our research reveals that the results of these risk algorithms vary widely in the population examined. Out of the ten different scores that were evaluated in this contemporary large cohort, the ASCVD showed the best agreement between calculated and observed risk.

An important, but not the sole reason for the differences between the scores are the variable components of the endpoints predicted. The strongest differences exist between the ESC-HS (indicates the risk of fatal cardiovascular events only), and the other algorithms which include nonfatal events. In the highest risk category of the ESC-HS, we did not observe a single fatal cardiovascular event in the age group (40–65 years) in which the algorithm has been developed. Therefore, we provisionally expanded the age range to 40 to 79 years, which resulted in improved comparability with other risk calculators, but the results need to be interpreted with caution.

The other algorithms also differ significantly in their calibration. FRS-CVD, FRS-CHD2, FRS-CHD1 and ARRIBA yield high results for the risk of broadly defined clinical endpoints; FRS hard-CVE, ASCVD, PROCAM I, PROCAM II, and Reynolds, which focus on narrowly defined clinical endpoints, provide lower results. These differences are not exclusively based on different endpoints. For instance, the end-points of ARRIBA and ASCVD are similar, but ARRIBA yields significantly higher risks.

For broad clinical endpoints the discriminatory power of all algorithms was lower than for narrowly defined endpoints, and it was surprisingly irrelevant what endpoint was originally used for the creation of a score. No significant differences were seen when the discrimination of all algorithms for a single endpoint was calculated. The reason for this observation could be that so-called “soft” end-points are less reliably detected and annotated. For instance, the end-point angina pectoris may be diluted with noncardiac chest pain. In addition to the end-point definition, the clinical parameters that define the risk formulas vary. Age, gender, smoking status, and systolic blood pressure are included in all risk equations, but cholesterol (total or LDL or HDL cholesterol), diabetes mellitus status, family history, antihypertensive therapy, HbA1c and CRP are only used in part of them.

The risk calculators correlate well with each other, but at high risks ARRIBA shows a strong upward deviation and is thus not optimally calibrated in this range, i.e. the predicted risks are significantly higher than the observed ones. Reynolds, FRS-CHD2 and ASCVD seem to be more accurate at higher risks. Allan *et al*.^22^ have come to a similar result: They compared 25 different risk calculators in 128 hypothetical patients with maximal seven risk factors. The absolute risks differed most at calculated risks above 20%. They conclude that this is of secondary importance for clinical decisions because all risk categories above 20% will be classified as “high”, regardless whether the calculated risk is 30%, 50% or 70%.

When we calculated continuous net reclassification improvements according to Pencina *et al*.^21^ 18 out of the 36 pairwise comparisons revealed statistically different reclassifications of subjects which also indicated substantial differences between the scores.

Rose^28^ points out that the focus of preventive strategies on a limited number of persons with putatively high risk has great benefit individually, but a smaller effect on the incidence rate of events in a population overall. Because most cardiovascular events occur in individuals with apparently low risk they are excluded from preventive treatment if a “high risk strategy” is followed stringently. For this reason, we have considered three different scenarios with different thresholds for “high risk” (and thus treatment recommendation) (Table 4).

At the intervention threshold of 20% in 10 years currently recommended in many guidelines 50 to 80% of patients who will suffer a cardiovascular event in the next 10 years will be excluded from therapeutic interventions, since they are not classified as high-risk patients. On the other hand, if the intervention threshold is lowered to 5%, the sensitivity greatly increases, so that more than 90% of all patients with subsequent cardiovascular events will be identified. However, this is expected to be at the expense of specificity and many patients who will never experience an event would also receive a therapy with the potential risk of side effects of medications or lower quality of life.

At the intervention threshold of 10% and 2.5%, respectively, in 10 years, ASCVD, FRS-CVD, ARRIBA, and ESC-HS have the highest sensitivities (74 to 84%). The specificities (69% and 64%) and the predictive value of positive tests (18% and 14%, proportion of persons with positive tests, in which an event also occurs) are highest for the ASCVD and the ARRIBA. The ESC-HS has a very low predictive value of positive tests (5%) but a high specificity of 74%.

Due to the good calibration over the entire range of risks, the large age range and the combined fatal and non-fatal endpoint, we conclude that the ASCVD is the preferable risk score for Germany. Using the ASCVD, the threshold of a 10-years risk of 10% is exceeded by one-third of the study participants older than 40 years. The ASCVD is based on the very recent studies ARIC (Atherosclerosis Risk in Communities)^29^, the Cardiovascular Health study^30^, the CARDIA (Coronary Artery Risk Development in Young Adults) study^31^, and the Framingham study^32,33^ and calculates the probability of a non-letal myocardial infarction, a letal or non-letal stroke or death due to coronary heart disease^34^. Finally, our data do not confirm the reported overestimation of the risks by the ASCVD in other cohorts^23–25,35^.

The proposed risk threshold of 10% is very close to the threshold of 7.5% recommended by the national US guidelines for intervention with moderate to high intensity statins treatment in primary prevention^2^. A most recent published study of the Copenhagen General Population showed that the application of lower risk thresholds for statin therapy could prevent more atherosclerotic cardiovascular events than the use of higher thresholds for therapy and intervention^36^.

Using the ASCVD, 30% of our cohort would require treatment. Assuming an event rate of 17% in 10 years and a relative risk reduction of 30% as an effect of treatment, the number needed to treat with statins and antihypertensive drugs to prevent one event would be reasonable at about 20 in 10 years or 40 in 5 years.

### Limitations

#### Absolute incidence rate of events

The total number of events recorded during follow up was comparatively low. This is related to the fact that we strictly confined our evaluation to a primary care population free of vascular disease at baseline and that the duration of the follow-up was limited. However, the absolute incidence rate of vascular events appears to be within the range of other cohorts recruited in Germany^37–39^.

#### Representativeness for primary care in Germany

Eligible doctors were identified to evenly represent the geographic areas of Germany at high granularity. The overall response rate of 60.2% was lower than in other studies with nation-wide random sampling^40,41^. This lower participation rate may be due to fact that eligible doctors were asked beforehand for their willingness to step into the more demanding third laboratory and follow-up layer of the study. Yet, in comparison to a study using a similar sampling strategy^40^ we did not identify any selective drop-outs by region or type of primary care setting. Further, 90% of patients eligible also participated.

Comparisons of our study sample to the entire DETECT population and the laboratory sample (third layer) revealed significant differences, because individuals with cardiovascular disease and diabetes mellitus were excluded from the current analysis (Supplementary Table 1). We also used only a part of the laboratory sample in which all items needed to calculate the risk scores were available. This subgroup and the laboratory cohort (third layer, Supplementary Tables 1 and 2) free of cardiovascular disease and diabetes mellitus were not significantly different from each other. Taken together, we are convinced that our study population is representative of the corresponding population in Germany. This is also exemplified by the prevalence rate of hypertension in the DETECT study which corresponds to the ones in a series of other German studies, even more recent ones (Supplementary Table 4)^39,42,43^. For further details we refer to a previous article addressing the representativeness of the DETECT study^44^.

#### Medication use

The degree at which medication may have confounded our results is hard to estimate. It needed to be considered that (a) the use of anti-hypertensives is already part of some of the risk scores while it may influence all of them by affecting blood pressure values at baseline or during follow-up (Table 2) (b) that hypertension was under-treated (Table 1) (c) that anti-thrombotic and lipid-lowering treatment had been prescribed to a low proportion of patients only (Table 2).

#### Use of the ASCVD in Germany

Differences in CVD risk have been reported between East and West Germany^45,46^ most likely as a sequel of differences in life-style, nutrition and social systems before the reunion of Germany. Very recently, however, the living conditions in the former East and West have been converging. It further needs to be considered, that the nature and the strength of an association between a risk factor and clinical endpoints is unlikely to differ between East and West Germany. Rather the prevalence rate and expression of risk factors had likely been responsible for the differences in cardiovascular disease burden between the two geographical areas of Germany in the past. Unexpectedly, we found that the ASCVD is well suited to German primary care. Apart from Caucasians, the ASCVD has included persons of Hispanic and African-Americans. These ethnicities are hardly represented in Germany, while an inclusion of Arab and Turkish immigrants is currently emerging. None of the algorithms examined here allows adjustment for these ethnicities nor is there any data available that would allow for taking this demographic change into account.

## Conclusion

In conclusion, the ASCVD (pooled cohort equation) recommended in the US guidelines is well suitable for Germany. At an intervention threshold of 10% risk in 10 years the ASCVD has a favorable ratio of sensitivity (80%) and specificity (about 70%), it combines non-fatal and fatal events as endpoint and can be applied over a wide age range.

## Methods

### Study design, participants and clinical characterization

The DETECT study has been a three-layer, multi-center, prospective long-term study and was initiated to investigate the prevalence and time course of CHD and its metabolic risk factors in primary care patients in Germany. Details of the study protocol have been published^44^. The study had been reviewed and approved by the Ethics Committee of the Medical Faculty Carl Gustav Carus at the Technical University Dresden (AZ: EK149092003; 16.09.2003) and registered at clinicaltrials.gov (NCT01076608). All participants were informed about the study and gave written informed consent. The authors confirm that all research was performed in accordance with relevant guidelines/regulations, the “Declaration of Helsinki” and the German data protection rules in place at the time of conducting the study.

The first layer was the recruitment of centers which was based on a nation-wide sample of physicians with primary care functions (medical practitioners, general practitioners, general internists). Sampling was based on 1060 regional segments (according to the criteria of IQVIA, formerly the Institute for Medical Statistics, Frankfurt am Main, Germany), clustered into 128 geographical areas for which primary care practitioners’ addresses were available. From this database a random sample of 7053 physicians was drawn. A total of 468 study monitors was responsible for recruiting these doctors. Monitors were requested to inform doctors about the study aims and procedures, to recruit up to eight doctors, strictly following the order on the list provided and to collect reasons not to participate. Out of initially 7053 eligible primary care physicians, 3188 (45.2%) finally joined in. The most common reasons for non-participation were: protocol too sophisticated, no interest, no participation in clinical trials in general, allowance not high enough, ethical concerns, lack of time or at baseline not available.

On the second layer, the participating physicians were instructed to screen all patients presenting in their practice alternatively on the forenoon of either the 16th or 18th of September 2003. The protocol specifically demanded inclusion of all attendees and prohibited any systematic choice of patients to provide a typical reflection of their everyday practice and avoid major bias. Exclusion criteria for the patients were: age under 18 years, the presence of a life-threatening illness, dementia or other serious, cognitive disorders, severe visual limitations. The total number of eligible patients was 59,403 patients to whom questionnaires were distributed. 3607 patients refused participation. In an additional 278 patients no doctor’s assessment was performed, leaving a total number of 55,518 patients (response rate 93.5%) for the DETECT main investigation.

For the third layer, 1000 doctors of the main study were randomly selected for participation in the laboratory and follow-up arm of DETECT. Participating doctors in this arm were asked to additionally include at least 12 randomly selected patients to undergo laboratory analysis and follow-up investigation. In 7521 patients the laboratory screening program was completed, valid laboratory data were obtained for a total 7519 patients from 851 doctors.

At each visit, physicians documented symptoms, diagnoses, treatments and health behavior of patients due to a structured interview; current heart rate, body mass index, waist and hip circumference and systolic and diastolic blood pressure were measured. Patients reported information about their health status and their psychosocial situation in a structured questionnaire.

### Laboratory testing

Blood samples were collected in the morning and sent to the Clinical Institute of Medical and Chemical Laboratory Diagnostics of the Medical University of Graz overnight. Cholesterol, triglycerides, glucose and “highly-sensitive” C-reactive protein (hsCRP) were determined on a Roche Modular automatic analyzer. LDL and HDL cholesterol were determined using a HELENA SAS-3/4-SAS electrophoresis system after separation of plasma proteins and enzymatic detection of cholesterol in lipoproteins by densitometry. Hemoglobin A1c (HbA1c) was measured on a ADAMS HA 8160 analysis system^44^.

### Clinical definitions

Hypertension was defined as systolic blood pressure >140 mm Hg, diastolic blood pressure >90 mm Hg^47^, a history of hypertension and/or the use of antihypertensive drugs. Diabetes mellitus was defined as glycated hemoglobin A1c (HbA1c) about 6.5% or fasting glucose above 125 mg/dl^48^, a history of diabetes mellitus and/or the use of oral hypoglycemic agents or insulin. Study participants were classified as active smokers, when they consumed a tobacco product in the last four weeks preceding the survey.

### Endpoints

One year and four years after the recruitment of patients the health status was documented by the participating investigators. Total mortality and cardiovascular causes of death, nonfatal MI, coronary revascularization (bypass surgery (CABG) or percutaneous coronary intervention), fatal and non-fatal stroke, transient cerebral ischemia, and symptomatic occlusive peripheral arterial disease were documented. The information about the endpoints was collected using a standardized form by the family physician and/or the facility in which the patient has previously been treated. The median time of follow-up was 4.02 years, the maximum period 4.6 years.

### Risk algorithms

We considered the following risk models: Framingham-hard-Cardiovascular Endpoints (FRS-hard-CVE)^11^, Framingham CHD1 (FRS-CHD1) and Framingham CHD2 (FRS-CHD2)^12^, Framingham CVD (FRS-CVD)^49^, ARRIBA (which is widely used by general practitioners in Germany), PROCAM I^38^ and PROCAM II^50^, Reynolds score^51,52^, ESC Heart Score (ESC-HS)^13^ and atherosclerotic cardiovascular disease score (ASCVD), sometimes called Pooled Cohort Equation^34^. The algorithm for the calculation of a continuous PROCAM score in its latest version of 2007^50^ is not public, since the supplemental data mentioned in the publication is not accessible. For this reason, in Fig. 2, Supplementary Figs 1 and 2, and in Table 3 and Supplementary Table 4, only the PROCAM I version published in 2002 was used in which the risk for women was estimated by dividing the calculated risk of men by 4.

The corresponding risks were calculated for each of the study participants based on the records of the first survey in 2003. Table 2 provides an overview of the covariates included in the risk algorithms. FRS-CHD1 and FRS-CHD2 differ because FRS-CHD1 uses total cholesterol and FRS-CHD2 uses LDL cholesterol. For the calculation of the ESC-HS, the risk algorithm was applied as proposed for Germany^13,53,54^. For ARRIBA the risks were determined by using accessible risk charts (www.arriba-hausarzt.de/material/papier.html).

### Sample and subgroups

In our analysis, we included patients from the third layer of the study in whom a) follow-up data (one year and four years after the start of the study) was available or who had died during the observation period, b) at recruitment no evidence of coronary artery disease, symptomatic peripheral arterial disease, cancer, severe kidney disease existed nor a history of heart attack or stroke, and c) no diabetes mellitus was diagnosed. Patients with diabetes mellitus were excluded because in relevant guidelines^55^ diabetes mellitus is treated as a “coronary risk equivalent” so that risk calculation would not be needed. After application of these criteria our sample consisted of 4044 patients.

Different inclusion criteria and different clinical endpoints were used through the development of the algorithms. We have adopted these criteria and defined five combined clinical endpoints (EP): EP1: fatal and non-fatal myocardial infarction, revascularization, sudden cardiac death (PROCAM I/II, FRS hard-CVE); EP2: EP1 plus fatal and non-fatal stroke (Reynolds, ASCVD, ARRIBA); EP3: EP1 plus angina (FRS CHD1, FRS CHD2); EP4: EP1 plus heart failure (NYHA III or IV), fatal and non-fatal stroke, transient ischemic attack (TIA), symptomatic peripheral arterial disease (PAD) (FRS-CVD); EP5: death by cardiovascular cause (ESC-HS). For further details see Table 2 and Fig. 1.

### Statistical methods

The characteristics of the study cohort are presented as means and standard deviations (continuous traits) and relative frequencies (categorial traits) (Table 1).

*Correlations* of 10-years risks at the first investigation in 2003 were calculated according to Spearman and Pearson (Supplementary Table 4), the relationship between the 10-years risks is shown in scatter plots with best-fit lines and their 95% confidence intervals, with the FRS-CVD on the abscissa. Both axes are scaled logarithmically, because the risk values obtained were skewed to the right in the study sample (Supplementary Fig. 1). Based on the Spearman correlation matrix the entries of which can be considered as “distances” between scores, a multidimensional scaling was performed (Supplementary Fig. 2). The lower the “distance” between two scores in the graph, the higher is their correlation.

#### Discrimination

To compare the discriminatory power of the prognostic models, we calculated the areas under the receiver operator characteristics curves (AUCs) and the Harrell’s C-statistics for all considered risk equations in a subpopulation of persons 40 to 65 years of age (n = 2463) (Table 3).

#### Calibration

To examine the concordance of the predicted with the incidence rates actually observed, we divided the subpopulations in risk groups <10%, 10–20%, and ≥20%. For each of the resulting risk groups, we plotted the means of the calculated risks against the 10-year rates of associated endpoints (Fig. 3). The event rates were extrapolated to 10 years assuming that the cumulative incidence rate for the events is linear in time. Additionally, we have divided the calculated risks in deciles and again compared the mean predicted risks with the actual incidence rates. For each of the risk groups, (“low” to “very high”), we calculated the 95% confidence intervals. We examined how well the risk score approximates the observed incidence rates in the score deciles using the Hosmer-Lemeshow test. Specifically: If K_j_ is the number of observed events in the j-th decile with n observations, E_j_ is the expected sum of the events and Σ is the test statistic from the sum of H_j_ = (K_j_ − E_j_)^2^/E_j_(1 − E_j_/n), j = 1, …, 10, then the p-value is equal to the chi-square function with eight degrees of freedom in Σ (Table 4). Sensitivity, specificity, positive and negative predictive value of the algorithms at the threshold values 5, 10 and 20% of the calculated risks are given in Table 5. All calculations were performed using R (R-Project, version 3.1.3) and Matlab (version R2015a). Continuous net reclassification improvements (Supplementary Table 5) were calculated according as described^21^.

## Supplementary information

Dataset 1
