# Author Correction: Cardiovascular risk algorithms in primary care: Results from the DETECT study

**DOI:** 10.1038/s41598-020-59763-0

**Published:** 2020-03-31

**Authors:** Tanja B. Grammer, Alexander Dressel, Ingrid Gergei, Marcus E. Kleber, Ulrich Laufs, Hubert Scharnagl, Uwe Nixdorff, Jens Klotsche, Lars Pieper, David Pittrow, Sigmund Silber, Hans-Ulrich Wittchen, Winfried März

**Affiliations:** 1 University of Heidelberg, Mannheim Medical Faculty, Mannheim Institute of Public Health, Social and Preventive Medicine, Mannheim, Germany; 2 University of Heidelberg, Mannheim Medical Faculty, Department of Internal Medicine V (Nephrology, Hypertensiology, Rheumatology, Endocrinology, Diabetology), Mannheim, Germany; 3 Clinic and Polyclinic of Cardiology, University Clinic Leipzig, Leipzig, Germany; 4 Medical University of Graz, Clinical Institute of Medical and Chemical Laboratory Diagnostics, Graz, Austria; 5 European Prevention Center, EPC GmbH, Düsseldorf, Germany; 6 German Research Center of Rheumatology Berlin, Leibnitz Institute, Berlin, Germany; 7 Charité Universitätsmedizin Berlin, Institute of Social Medicine, Epidemiology and Health Economics, Berlin, Germany; 8 Technical University Dresden, Medical Faculty, Institute of Clinical Pharmacology, Dresden, Germany; 9 Cardiology Outpatient Clinic Tal, Munich, Germany; 10 Technical University Dresden, Institute of Clinical Psychology and Psychotherapy, Dresden, Germany; 11 Max-Planck- Institute of Psychiatry, Munich, Germany; 12 Synlab Services GmbH, Synlab Academy, Mannheim, Augsburg, Germany

Correction to: *Scientific Reports* 10.1038/s41598-018-37092-7, published online 31 January 2019

The Acknowledgements section in this Article is incomplete.

“We thank the participants, the study team of the DETECT study, and the laboratory staff at Graz. This work was supported by a grant from the German Federal Ministry of Economics and Energy to the University of Heidelberg, project Coropredict® (grant number 03EFBBWO58). The DETECT (Diabetes Cardiovascular Risk evaluation: Targets and Essential Data for Commitment of Treatment) - study was supported by an unrestricted educational grant from Pfizer GmbH, Karlsruhe, Germany. The sponsors of the study had no influence on the design, analysis or interpretation of data.”

should read:

“We thank the participants, the study team of the DETECT study, and the laboratory staff at Graz. This work was supported by a grant from the German Federal Ministry of Economics and Energy to the University of Heidelberg, project Coropredict® (grant number 03EFBBWO58). The DETECT (Diabetes Cardiovascular Risk evaluation: Targets and Essential Data for Commitment of Treatment) - study was supported by an unrestricted educational grant from Pfizer GmbH, Karlsruhe, Germany. The sponsors of the study had no influence on the design, analysis or interpretation of data. We acknowledge financial support by Deutsche Forschungsgemeinschaft within the funding programme Open Access Publishing, by the Baden-Württemberg Ministry of Science, Research and the Arts and by Ruprecht-Karls-Universität Heidelberg.”

