# Supplementary material for: Cardiovascular risk algorithms in primary care: Results from the DETECT study
Source: Sci Rep. 2019 Jan 31;9:1101. doi: 10.1038/s41598-018-37092-7 (PMC6355969; doi:10.1038/s41598-018-37092-7)
Supplement: Supplementary file 1 — Dataset 1 [file 41598_2018_37092_MOESM1_ESM.docx]

**Cardiovascular risk algorithms in primary care.**

**Results from the DETECT study**

Tanja B. Grammer, MD^1,2*^, Alexander Dressel, PhD^2^, Ingrid Gergei^2^, Marcus E. Kleber, PhD^2,3^, Ulrich Laufs, MD^4^, Hubert Scharnagl, PhD^5^, Uwe Nixdorff, MD^6^, Jens Klotsche, PhD^7^, Lars Pieper, PhD^8^, David Pittrow, PhD^9^, Sigmund Silber, MD^10^, Hans-Ulrich Wittchen, MD^11,12^, Winfried März, MD^2,5,13^

^1^University of Heidelberg, Mannheim Medical Faculty, Mannheim Institute of Public Health, Social and Preventive Medicine, Mannheim, Germany
^2^University of Heidelberg, Mannheim Medical Faculty, Department of Internal Medicine V (Nephrology, Hypertensiology, Rheumatology, Endocrinology, Diabetology), Mannheim, Germany
^3^Friedrich-Schiller-Universität Jena, Institute of Nutrition Science, Jena, Germany
^4^Clinic and Polyclinic of Cardiology, Univesity Clinic Leipzig, Leipzig, Germany
^5^Medical University of Graz, Clinical Institute of Medical and Chemical Laboratory Diagnostics, Graz, Austria
^6^European Prevention Center, EPC GmbH, Düsseldorf, Germany
^7^German Research Center of Rheumatology Berlin, Leibnitz Institute, Berlin, Germany

^8^Charité Universitätsmedizin Berlin, Institute of Social Medicine, Epidemiology and Health Economics, Berlin, Germany
^9^Technical University Dresden, Medical Faculty, Institute of Clinical Pharmacology, Dresden, Germany
^10^Cardiology Outpatient Clinic Tal, Munich, Germany
^11^Technical University Dresden, Institute of Clinical Psychology and Psychotherapy, Dresden, Germany
^12^Max-Planck- Institute of Psychiatry, Munich, Germany
^13^Synlab Services GmbH, Synlab Academy, Mannheim and Augsburg, Germany

**Supplementary Information**

**Supplementary Figure 1**

Comparison of risk algorithms by scatterplots. Based on the results of the first cross-sectional study of the DETECT population 10-years risks for each patient in the sample (n = 2463, subgroup aged 40-65 years) were calculated with all specified algorithms.

Abscissa: Framingham CVD (clinical endpoints: coronary death, myocardial infarction, coronary insufficiency, angina pectoris, heart failure, stroke, transient cerebral ischemia, peripheral arterial occlusive disease, EP4).

Ordinates: A) PROCAM I (clinical endpoint: coronary death, myocardial infarction, EP1); B) Reynolds (clinical endpoint: myocardial infarction, stroke, coronary revascularization, cardiovascular death, EP2); C) Framingham hard-CE (clinical endpoint: coronary death, myocardial infarction, EP1); D) Framingham CHD1 (clinical endpoint: coronary death, myocardial infarction, coronary insufficiency, angina pectoris, heart failure, EP3); E) Framingham CHD2 (clinical endpoint as Framingham CHD1); F) ARRIBA (clinical endpoint: myocardial infarction, stroke, coronary revascularization, cardiovascular death, EP2); G) ASCVD (clinical endpoint: myocardial infarction, stroke, coronary revascularization, cardiovascular death, EP2); H) ESC HeartScore (clinical endpoint: cardiovascular mortality, EP5).

If risks were derived from tables and these risks assumed discrete values ony, several patients fall together in one data point. PROCAM II is not shown here, since the continuous risk formula is not published and the score derived from the panels can assume five levels only.

**Supplementary Figure 2**

Embedding the scores in the three-dimensional space by means of multidimensional scaling. Based on the Spearman correlation matrix the items of which can be construed as "distances" between the scores, a multidimensional scaling was carried out; the projections of the "score points" in a three-dimensional space, spanned by the vectors to the three largest values of the Spearman correlation matrix were performed. The smaller the "distance" is between two scores the more similar they are.

**Supplementary Table 1. Characteristics of the entire third layer laboratory subgroup of the DETECT study and of the subpopulation without CVD examined in the current study**

| **Variable** | **Third layer**  **laboratory subgroup** | **Current**  **study population** **w/o CVD and DM** | **P^2^** |
| --- | --- | --- | --- |
| Number | 7519 | 4044 |  |
| Female, n (%) | 4438 (59) | 2641 (65.3) | <0.001 |
| Age (years) | 57.7±14.4 | 53.8±13.7 | <0.001 |
| HDL cholesterol (mg/dl) | 54.4±18.7 | 57.2±18.8 | <0.001 |
| LDL cholesterol (mg/dl) | 127.5±33.9 | 129.3±33.3 | <0.001 |
| Cholesterol (mg/dl) | 223.2±43.3 | 225.9±41 | <0.001 |
| Triglycerides (mg/dl)^1^ | 128.4 (72.5,227.2) | 118.7 (68.5,205.8) | <0.001 |
| HbA1c (%) | 5.6±0.9 | 5.3±0.3 | <0.001 |
| Glucose (mg/dl) | 114.7±39.6 | 101.7±16.3 | <0.001 |
| hsCRP (mg/l)^1^ | 2.3 (0.7,7.1) | 2 (0.7,6.4) | <0.001 |
| Arterial hypertension, n (%) | 3615 (49) | 1502 (37.14) | <0.001 |
| Systolic blood pressure (mm Hg) | 132.6±18.6 | 129.5±17.5 | <0.001 |
| Diastolic blood pressure (mm Hg) | 80.1±10 | 79.6±9.8 | <0.001 |
| Smoking never/ex/current (%) | 3994/1734/1519 (55.1/23.9/21) | 2291/819/934 (56.7/20.3/23.1) | <0.001 |
| Positive family history, n (%) | 2207 (30.4) | 1082 (26.8) | <0.001 |
| BMI (kg/m²) | 27.2±4.9 | 26.4±4.6 | <0.001 |
| **Medication** |  |  |  |
| Antihypertensive medication, n (%) | 2821 (37.5) | 1086 (26.9) | <0.001 |
| Lipid lowering medication, n (%) | 1134 (15.1) | 313 (7.7) | <0.001 |
| Antithrombotics, n (%) | 1121 (14.9) | 226 (5.6) | <0.001 |
| Antidiabetic medication n, (%) | 979 (13) | 0 (0) | <0.001 |
| **Clinical endpoints during follow-up** |  |  |  |
| Death, n (%) | 279 (3.7) | 75 (1.9) | <0.001 |
| Cardiovascular Death, n (%) | 210 (2.8) | 57 (1.4) | <0.001 |
| Non letal myocardial infarctions, n (%) | 85 (1.1) | 19 (0.5) | <0.001 |
| PTCA/ACVB, n (%) | 186 (2.5) | 36 (0.9) | <0.001 |
| Stroke, TIA, n (%) | 189 (2.5) | 69 (1.7) | <0.001 |
| Symptomatic periperal arterial disease, n (%) | 112 (1.5) | 32 (0.8) | <0.001 |
| Endpoint 1 (PROCAM-I, FRS-hard-CVE), n (%) | 319 (4.2) | 72 (1.8) | <0.001 |
| Endpoint 2 (Reynolds, ASCVD, ARRIBA), n (%) | 390 (5.2) | 101 (2.5) | <0.001 |
| Endpoint 3 (FRS-CHD1, FRS-CHD1), n (%) | 519 (6.9) | 132 (3.3) | <0.001 |
| Endpoint 4 (FRS-CVD), n (%) | 775 (10.3) | 220 (5.4) | <0.001 |

^1^ Values for triglycerides and hsCRP in brackets correspond to logarithmic standard intervals

^2^ Student´s T test and exact Fisher´s test in the case of continuous and categorial variables, respectively (by comparing the current study population with its complement in the third-layer laboratory subgroup)

**Supplementary Table 2. Characteristics of the third layer laboratory subgroup of the DETECT study without CVD and DM and of the subpopulation without CVD and DM examined in the current study**

| **Variable** | **Third layer**  **laboratory subgroup w/o CVD and DM** | **Current**  **study population** **w/o CVD and DM** | **P^2^** |
| --- | --- | --- | --- |
| Number | 4473 | 4044 |  |
| Female, n (%) | 2927 (65.4) | 2641 (65.3) | 0.594 |
| Age (years) | 53.9±13.8 | 53.8±13.7 | 0.306 |
| HDL cholesterol (mg/dl) | 57.2±18.7 | 57.2±18.8 | 0.719 |
| LDL cholesterol (mg/dl) | 129.5±33.3 | 129.3±33.3 | 0.319 |
| Cholesterol (mg/dl) | 226.1±41.2 | 225.9±41 | 0.456 |
| Triglycerides (mg/dl)^1^ | 118.9 (68.6,206.1) | 118.7 (68.5,205.8) | 0.589 |
| HbA1c (%) | 5.3±0.3 | 5.3±0.3 | 0.761 |
| Glucose (mg/dl) | 101.8±16.4 | 101.7±16.3 | 0.064 |
| hsCRP (mg/l)^1^ | 2 (0.7,6.4) | 2 (0.7,6.3) | 0.425 |
| Arterial hypertension n (%) | 1647 (37.6) | 1502 (37.1) | 0.03 |
| Systolic blood pressure (mm Hg) | 129.6±17.6 | 129.5±17.5 | 0.575 |
| Diastolic blood pressure (mm Hg) | 79.6±9.8 | 79.6±9.8 | 0.571 |
| Smoking never/ex/current (%) | 2455/861/1006 (56.8/19.9/23.3) | 2291/819/934 (56.7/20.3/23.1) | 0.093 |
| Positive family history, n (%) | 1163 (26.7) | 1082 (26.8) | 0.894 |
| BMI (kg/m²) | 26.4±4.6 | 26.4±4.6 | 0.879 |
| **Medication** |  |  |  |
| Antihypertensive medication, n (%) | 1197 (26.8) | 1086 (26.9) | 0.688 |
| Lipid lowering medication, n (%) | 350 (7.8) | 313 (7.7) | 0.508 |
| Antithrombotics, n (%) | 240 (5.4) | 226 (5.6) | 0.042 |
| **Clinical endpoints during follow-up** |  |  |  |
| Death, n (%) | 87 (2) | 75 (1.9) | 0.195 |
| Cardiovascular Death, n (%) | 68 (1.5) | 57 (1.4) | 0.092 |
| Non letal myocardial infarctions, n (%) | 20 (0.5) | 19 (0.5) | 0.715 |
| PTCA/ACVB, n (%) | 40 (0.9) | 36 (0.9) | 0.79 |
| Stroke, TIA, n (%) | 75 (1.7) | 69 (1.7) | 0.843 |
| Symptomatic periperal arterial disease, n (%) | 33 (0.7) | 32 (0.8) | 0.365 |
| Endpoint 1 (PROCAM-I, FRS-hard-CVE), n (%) | 79 (1.8) | 72 (1.8) | 1 |
| Endpoint 2 (Reynolds, ASCVD, ARRIBA), n (%) | 113 (2.5) | 101 (2.5) | 0.63 |
| Endpoint 3 (FRS-CHD1, FRS-CHD1), n (%) | 154 (3.4) | 132 (3.3) | 0.051 |
| Endpoint 4 (FRS-CVD), n (%) | 249 (5.6) | 220 (5.4) | 0.267 |

^1^ Values for triglycerides and hsCRP in brackets correspond to logarithmic standard intervals

^2^ Student´s T test and exact Fisher´s test in the case of continuous and categorial variables, respectively (by comparing the current study population with its complement in the third-layer laboratory subgroup)

**Supplementary Table 3. Characteristics of study participants living in East and West Germany.**

| **Variable** | **East Germany** | **West Germany** | **P^3^** |
| --- | --- | --- | --- |
| Number | 1253 | 2791 |  |
| female, n (%) | 836 (66.7) | 1805 (64.7) | 0.211 |
| Age (years) | 54.1±13 | 53.7±14.1 | 0.358 |
| HDL cholesterol (mg/dl) | 58±18.7 | 56.8±18.8 | 0.075 |
| LDL cholesterol (mg/dl) | 129.4±32.6 | 129.3±33.7 | 0.941 |
| Cholesterol (mg/dl) | 228.2±40.8 | 224.9±41 | 0.020 |
| Triglycerides (mg/dl)^3^ | 123.92 (71.1,215.9) | 116.43 (67.4,201.2) | 0.001 |
| HbA1c (%) | 5.3±0.3 | 5.3±0.3 | 0.087 |
| Glucose (mg/dl) | 102.8±16.9 | 101.2±16 | 0.005 |
| hsCRP (mg/l)^3^ | 2 (0.7,6.2) | 2.02 (0.6,6.4) | 0.807 |
| Arterial hypertension, n (%) | 498 (39.7) | 1004 (36) | 0.022 |
| Systolic blood pressure (mm Hg) | 129.1±16.4 | 129.7±17.9 | 0.251 |
| Diastolic blood pressure (mm Hg) | 79.6±8.8 | 79.6±10.2 | 0.914 |
| Smoking never/ex/current (%) | 768/246/239 (61.3/19.6/19.1) | 1523/573/695 (54.6/20.5/24.9) | <0.001 |
| Positive family history, n (%) | 355 (28.3) | 727 (26.1) | 0.134 |
| BMI (kg/m²) | 26.6±4.6 | 26.3±4.7 | 0.023 |
| **Medication** |  |  |  |
| Antihypertensive medication, n (%) | 395 (31.5) | 691 (24.8) | <0.001 |
| Lipid lowering medication, n (%) | 106 (8.5) | 207 (7.4) | 0.253 |
| Antithrombotics, n (%) | 75 (6) | 151 (5.4) | 0.46 |
| **Clinical endpoints during follow-up** |  |  |  |
| Death, n (%) | 20 (1.6) | 55 (2) | 0.452 |
| Cardiovascular Death, n (%) | 15 (1.2) | 42 (1.5) | 0.475 |
| Non letal myocardial infarctions, n (%) | 4 (0.3) | 15 (0.5) | 0.459 |
| PTCA/ACVB, n (%) | 11 (0.9) | 25 (0.9) | 1 |
| Stroke, TIA, n (%) | 22 (1.8) | 47 (1.7) | 0.896 |
| Symptomatic periperal arterial disease, n (%) | 8 (0.6) | 24 (0.9) | 0.567 |
| Endpoint 1 (PROCAM-I, FRS-hard-CVE), n (%) | 18 (1.4) | 54 (1.9) | 0.305 |
| Endpoint 2 (Reynolds, ASCVD, ARRIBA), n (%) | 28 (2.2) | 73 (2.6) | 0.515 |
| Endpoint 3 (FRS-CHD1, FRS-CHD1), n (%) | 44 (3.5) | 88 (3.2) | 0.566 |
| Endpoint 4 (FRS-CVD), n (%) | 67 (5.4) | 153 (5.5) | 0.94 |

^1^ includes the federal states Berlin, Brandenburg, Mecklenburg-Western Pomerania, Saxony, Saxony-Anhalt, Thuringia

^2^ includes the federal states Baden-Württemberg, Bavaria, Bremen, Hamburg, Hessen, Lower Saxony, North Rhine-Westphalia, Rhineland-Palatinate, Saarland, Schleswig-Holstein

^3^ Values for triglycerides and hsCRP in brackets correspond to logarithmic standard intervals

^4^ Student´s T test and exact Fisher´s test in the case of continuous and categorial variables, respectively

**Supplementary Table 4. Correlations between 10-year risks for cardiovascular events, based on the first visit results of the DETECT study for participants 40-65 years old**

**(n=2463)**

|  | PROCAM-I | Reynolds | FRS-hard-CVE | FRS-CHD1 | FRS-CHD2 | FRS-CVD | ARRIBA | ASCVD | ESC-HS |
| --- | --- | --- | --- | --- | --- | --- | --- | --- | --- |
| PROCAM-I | 1 | **0.91** | **0.91** | 0.88 | 0.88 | **0.9** | 0.89 | 0.89 | 0.89 |
| Reynolds | **0.92** | **1** | **0.92** | **0.90** | **0.91** | **0.95** | 0.89 | **0.95** | **0.94** |
| FRS-hard-CVE | **0.93** | **0.92** | **1** | 0.87 | 0.89 | **0.94** | **0.95** | **0.96** | 0.89 |
| FRS-CHD1 | 0.89 | 0.89 | 0.86 | **1** | **0.98** | **0.92** | 0.83 | **0.90** | **0.9** |
| FRS-CHD2 | **0.90** | **0.90** | 0.88 | **0.97** | **1** | **0.93** | 0.85 | **0.91** | **0.91** |
| FRS-CVD | **0.90** | **0.95** | **0.94** | **0.92** | **0.93** | **1** | **0.90** | ***0.96*** | **0.92** |
| ARRIBA | **0.90** | 0.89 | **0.95** | 0.82 | 0.84 | **0.90** | **1** | **0.91** | 0.83 |
| ASCVD | **0.91** | **0.94** | **0.95** | 0.89 | **0.90** | **0.96** | **0.90** | **1** | **0.94** |
| ESC-HS | **0.90** | **0.94** | 0.88 | 0.88 | **0.9** | **0.92** | 0.82 | **0.94** | **1** |

Light grey: correlation coefficiants by Pearson

Dark grey: correlation coefficiants by Spearman

bold: correlation coefficiants greater or equal 0.90

**Supplementary Table 5. Pairwise continuous net reclassification improvements (NRI^1^) of risk scores (95% CI in brackets, significant NRIs bold)^2^**

|  | Reynolds | ASCVD | PROCAM-I | FRS-CHD1 | FRS-CHD2 | FRS-CVD | FRS-hard-CVE | ARRIBA | **ESC-HS** |
| --- | --- | --- | --- | --- | --- | --- | --- | --- | --- |
| Reynolds |  | 0.121 (-0.067,0.268) | **-0.157 (-0.282, -0.016)** | **-0.245  (-0.347, -0.061)** | **-0.173 (-0.323, 0)** | -0.039  (-0.164, 0.151) | -0.114  (-0.244, 0.027) | **-0.224 (-0.326, -0.105)** | 0.011 (-0.142, 0.183) |
| ASCVD | -0.121 (-0.268, 0.067) |  | -0.141 (-0.327, 0.004) | **-0.229 (-0.377, -0.076)** | **-0.206 (-0.353, -0.037)** | -0.104 (-0.227, 0.020) | **-0.219 (-0.356, -0.029)** | **-0.252 (-0.380, -0.109)** | **-0.145  (-0.266, -0.008)** |
| PROCAM-I | **0.157 (0.016, 0.282)** | 0.141 (-0.004, 0.327) |  | 0.013 (-0.137, 0.158) | 0.052 (-0.103, 0.166) | **0.174 (0.029, 0.321)** | 0.050 (-0.058, 0.194) | 0.070 (-0.087, 0.189) | 0.114 (-0.035, 0.268) |
| FRS-CHD1 | **0.245 (0.061, 0.347)** | **0.229 (0.076, 0.377)** | -0.013 (-0.158, 0.137) |  | 0.066 (-0.101, 0.261) | **0.306 (0.123, 0.447)** | **0.196 (0.060, 0.314)** | 0.029 (-0.119, 0.180) | **0.196 (0.007, 0.313)** |
| FRS-CHD2 | **0.173 (0, 0.323)** | **0.206 (0.037, 0.353)** | -0.052 (-0.166, 0.103) | -0.066 (-0.261, 0.101) |  | **0.268 (0.064, 0.430)** | **0.165 (0, 0.298)** | -0.024 (-0.164, 0.136) | 0.146 (-0.054, 0.322) |
| FRS-CVD | 0.039 (0.151, 0.164) | 0.104 (-0.020, 0.227) | **-0.174 (-0.321, -0.029**) | **-0.306 (-0.447, -0.123)** | **-0.268 (-0.430, -0.064)** |  | -0.095 (-0.245, 0.012) | **-0.210 (-0.315, -0.078)** | 0.015 (-0.131, 0.161) |
| FRS-hard-CVE | 0.114 (0.027, 0.244) | **0.219 (0.029, 0.356)** | -0.05 (-0.194, 0.058) | **-0.196 (-0.314, -0.06)** | **-0.165 (-0.298, 0)** | 0.095 (-0.012, 0.245) |  | **-0.160 (-0.270, -0.001)** | 0.085 (-0.080, 0.234) |
| ARRIBA | **0.224 (0.105, 0.326)** | **0.252 (0.109, 0.38)** | -0.07 (-0.189, 0.087) | -0.029 (-0.180, 0.119) | 0.024 (-0.136, 0.164) | **0.21 (0.078, 0.315)** | **0.16 (0.001, 0.270)** |  | **0.165 (0, 0.291)** |
| ESC-HS | -0.011 (0.183, 0.142) | **0.145 (0.008, 0.266)** | -0.114 (-0.268, 0.035) | **-0.196 (-0.313, -0.007)** | -0.146 (-0.322, 0.054) | -0.015 (-0.161, 0.131) | -0.085 (-0.234, 0.080) | **-0.165 (-0.291, 0)** |  |

^1^ adapted to competing risk prediction models with censored survival data (corresponding to the quantity defined as “1/2 NRI (>0)” in Pencina et al.^21^

^2^ in i^th^ row and j^th^ column: NRI of the j^th^ score in comparison to the i^th^ score on the age interval 20-75 years (n=3794), endpoint: union of EP 1-5 (171 events); median follow-up time: 4.02 years)

Supplementary Table 6. Comparison of prevalence rates of hypertension in German cohorts (%)

|  | DEGS1 (N=7096) | | | | | DETECT  (third layer population, N=7519) | | | | DETECT (N=4044, current study population w/o CVD and DM) | | | | | |  |
| --- | --- | --- | --- | --- | --- | --- | --- | --- | --- | --- | --- | --- | --- | --- | --- | --- |
|  | Men | | | Women | | Men | | Women | | | Men | | Women | | | |
| Age Stratum (years) | N | Prevalence rate (%) | | N | Prevalence rate (%) | N | Prevalence rate (%) | N | Prevalence rate (%) | | N | Prevalence rate (%) | N | Prevalence rate (%) | | |
| 18-29 | Not provided | 16.2 | | Not provided | 1.8 | 53 | 20.8 | 123 | 6.5 | | 32 | 21.9 | 88 | 8 | | |
| 30-39 | Not provided | 14.4 | | Not provided | 4.6 | 243 | 22.2 | 501 | 11.6 | | 159 | 18.2 | 376 | 9.84 | | |
| 40-49 | Not provided | 39.8 | | Not provided | 16.4 | 504 | 32.7 | 853 | 20.5 | | 339 | 25.1 | 677 | 18.9 | | |
| 50-59 | Not provided | 56.8 | | Not provided | 30.6 | 613 | 53.3 | 791 | 43.9 | | 329 | 43.5 | 532 | 39.1 | | |
| 60-69 | Not provided | 47.6 | | Not provided | 43.4 | 914 | 65.5 | 1065 | 60.9 | | 352 | 50.6 | 571 | 53.2 | | |
| 70-79 | Not provided | 61.3 | | Not provided | 65.6 | 601 | 71.7 | 784 | 71.3 | | 166 | 65.1 | 325 | 62.2 | | |
|  | HYDRA (N=45.093) | | | | | DETECT  (third layer population, N=7519) | | | | DETECT (N=4044, current study population w/o CVD and DM) | | | | |  |  |
|  | Men | | | Women | | Men | | Women | | | Men | | Women | | | |
| Age Stratum (years) | N | | Prevalence rate (%) | n | Prevalence rate (%) | N | Prevalence rate (%) | N | Prevalence rate (%) | | N | Prevalence rate (%) | N | Prevalence rate (%) | | |
| 16-29 | 385 | | 17.8 | 277 | 7.8 | 32 | 21.9 | 88 | 8 | | 32 | 21.9 | 88 | 8 | | |
| 30-44 | 1213 | | 32.5 | 1226 | 19.9 | 317 | 19.2 | 707 | 13.3 | | 317 | 19.2 | 707 | 13.3 | | |
| 45-59 | 2469 | | 57.6 | 2956 | 48.0 | 510 | 38.4 | 878 | 31.8 | | 510 | 38.4 | 878 | 31.8 | | |
| 60-74 | 4672 | | 73.6 | 5921 | 73.1 | 464 | 53.9 | 765 | 55.3 | | 464 | 53.9 | 765 | 55.3 | | |
| ≥75 | 1164 | | 76.1 | 2461 | 80.4 | 80 | 68.8 | 203 | 64 | | 80 | 68.8 | 203 | 64 | | |

**Supplementary Figure 1**


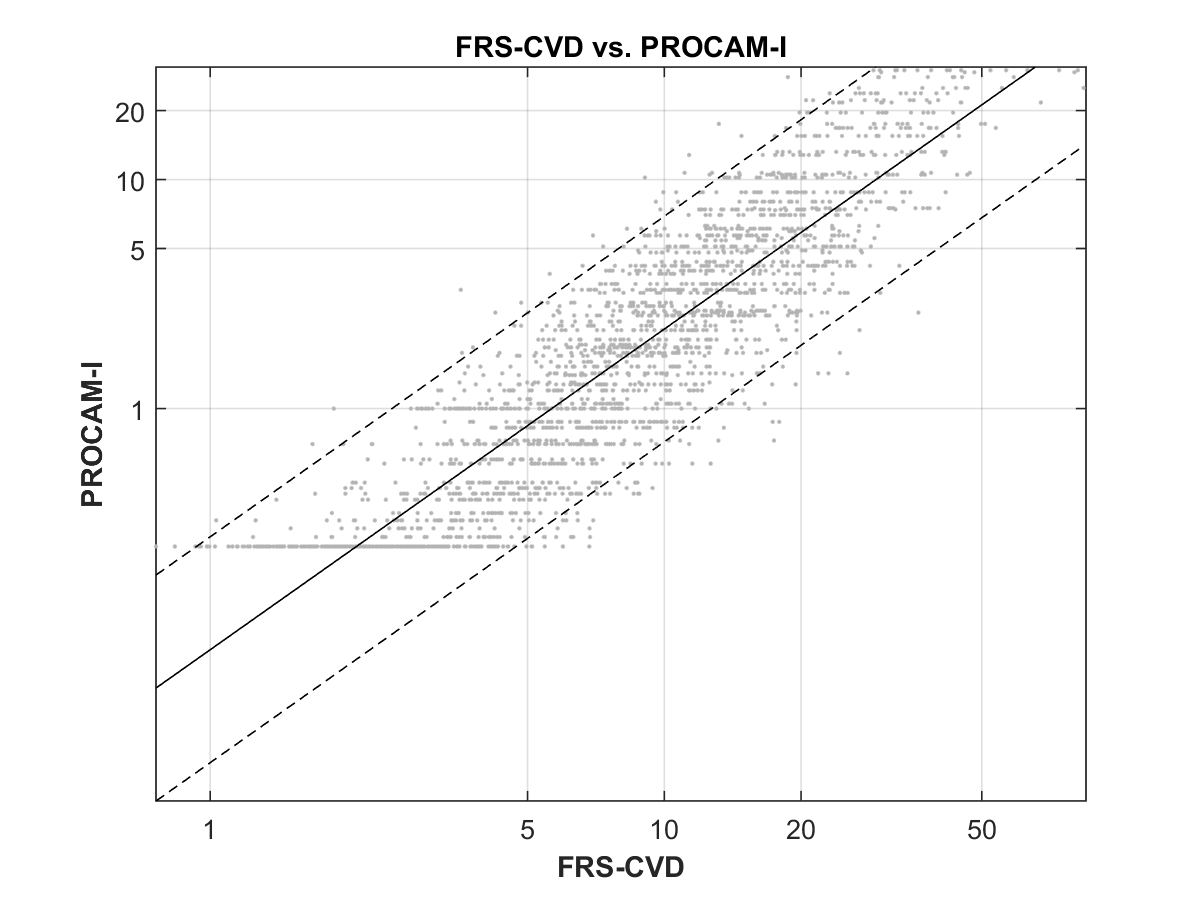

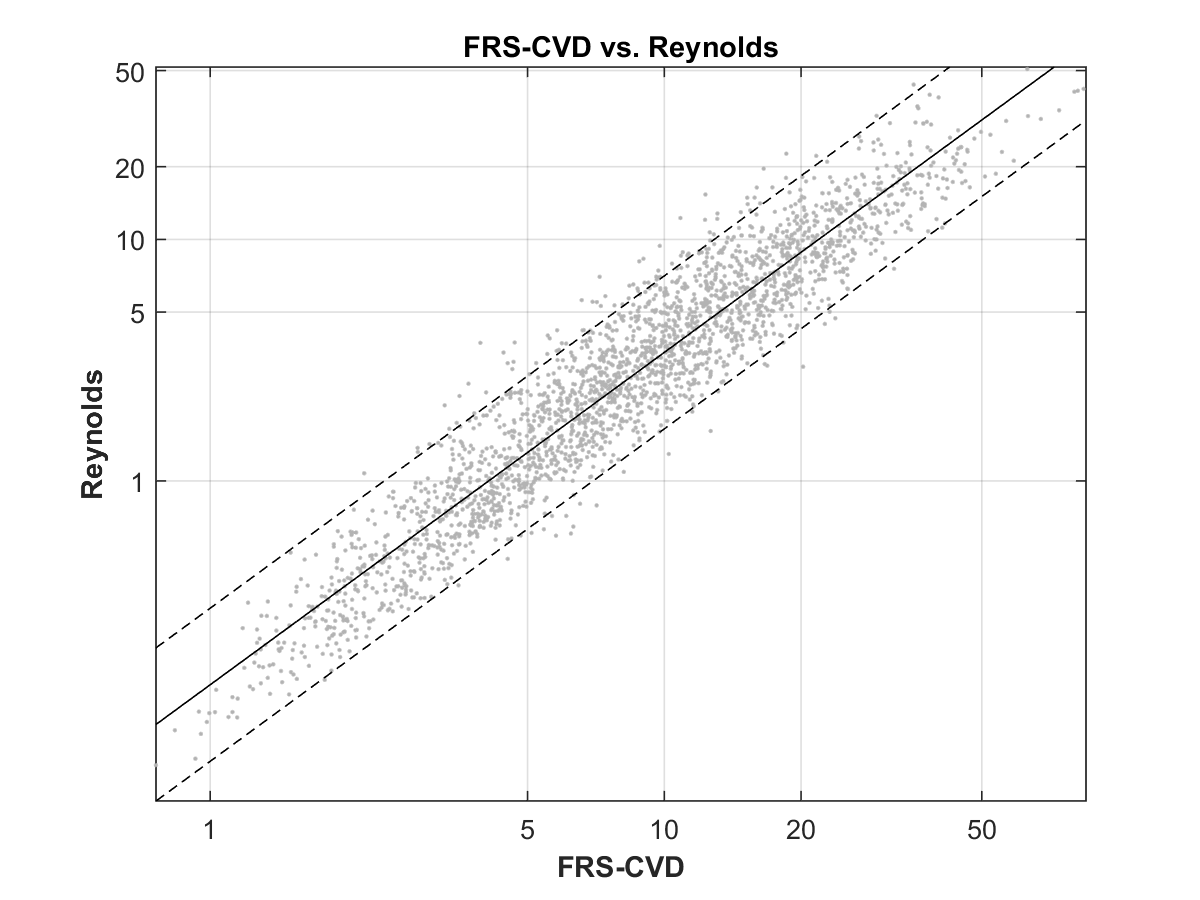

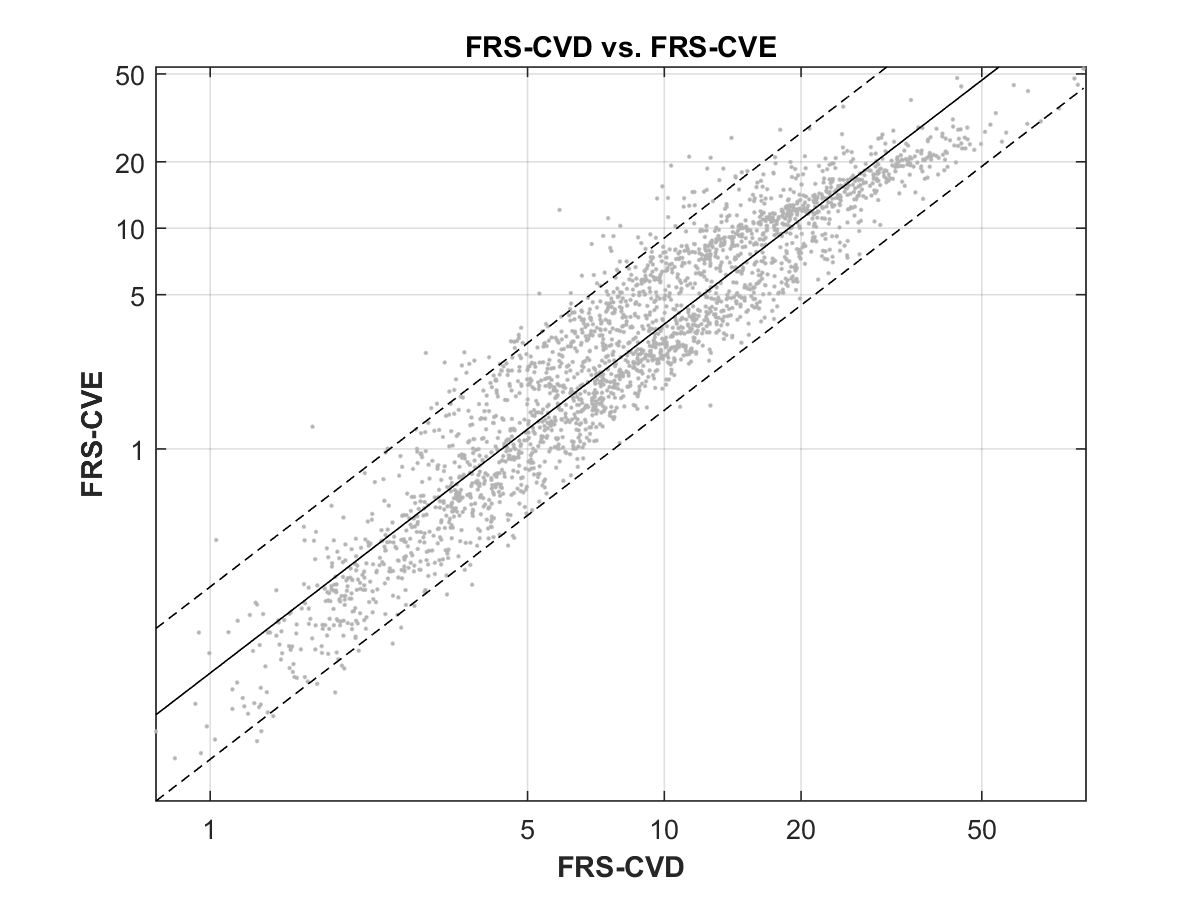

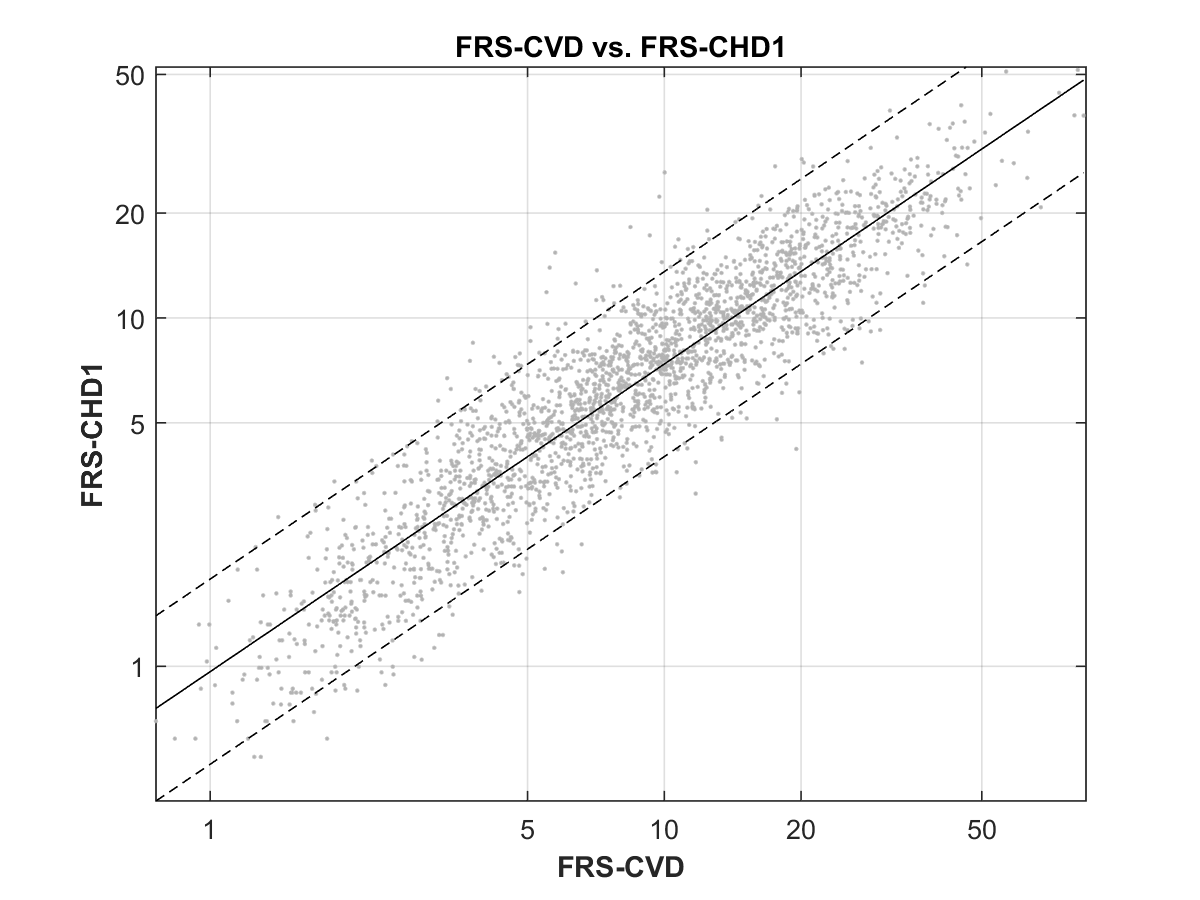

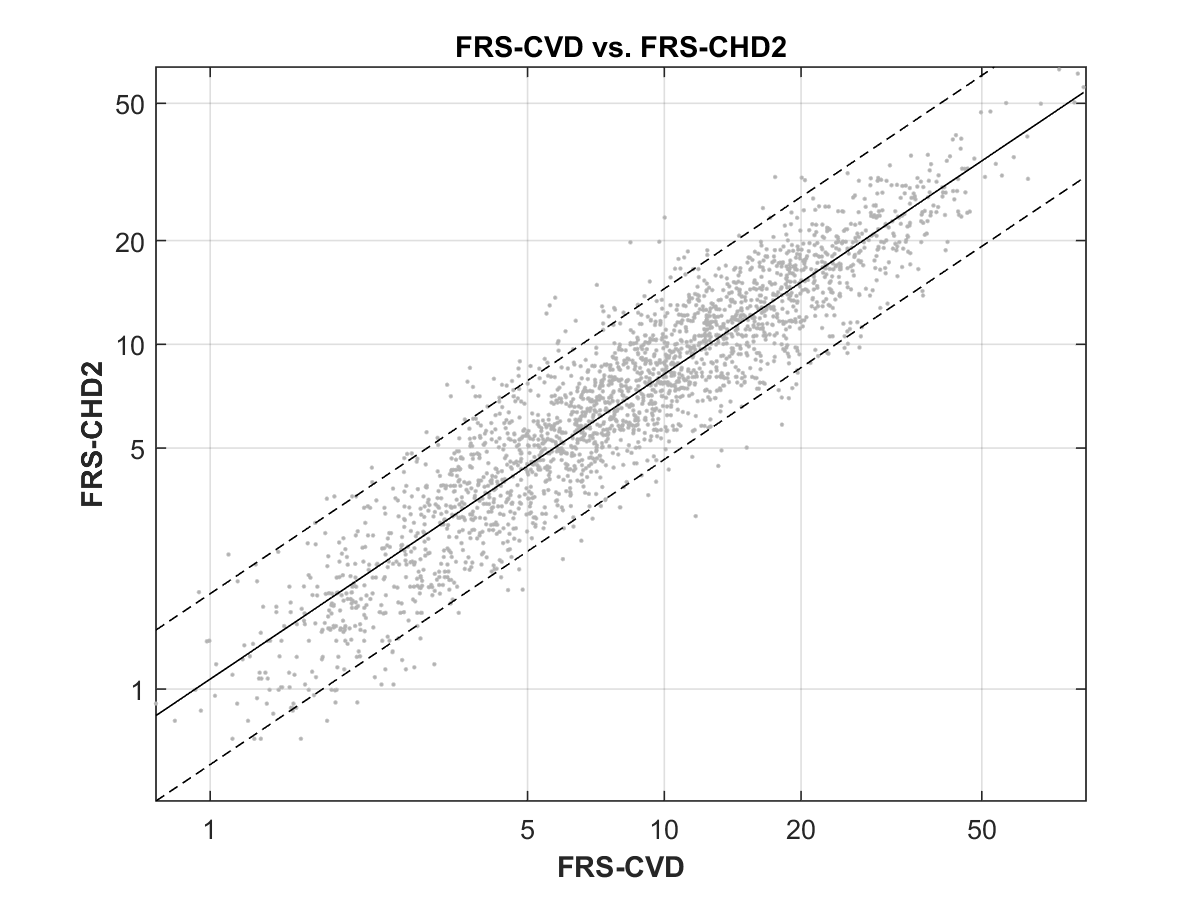

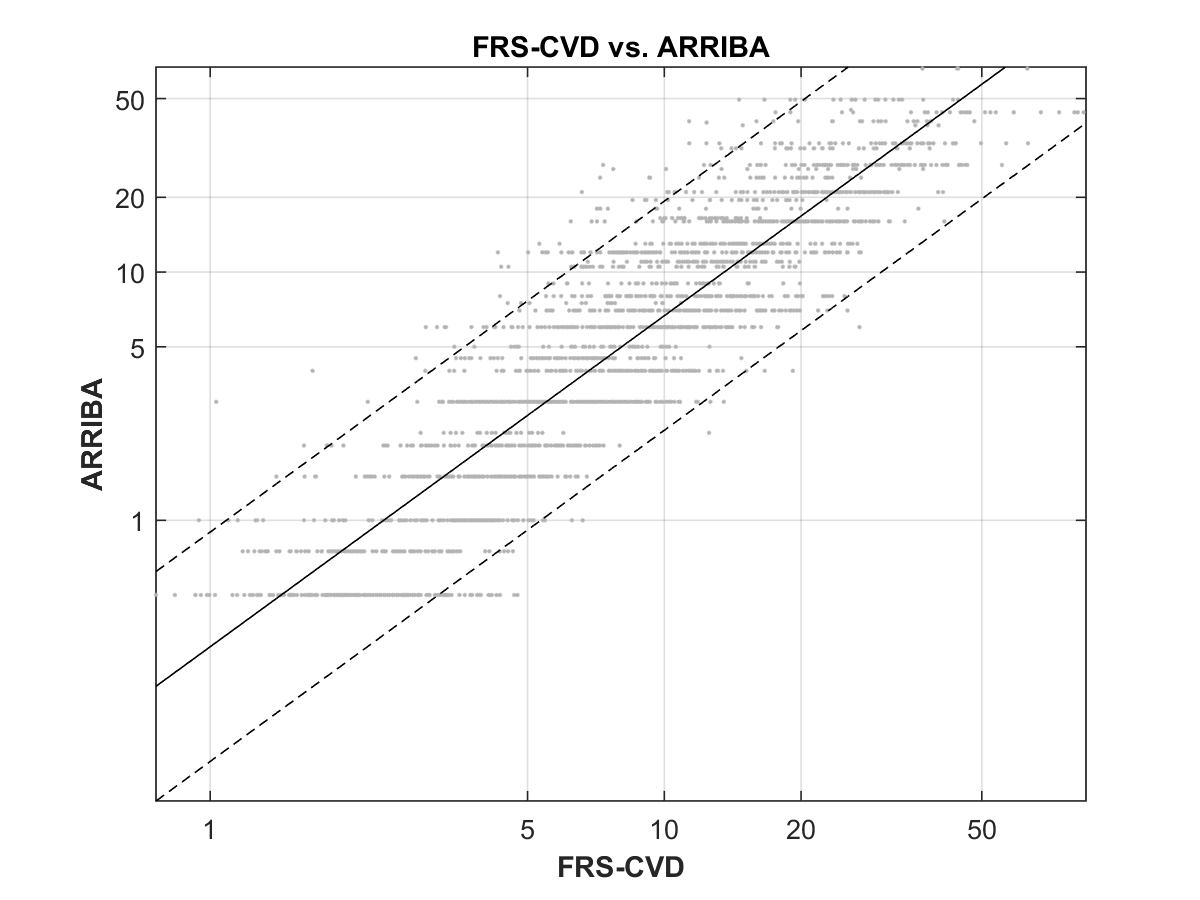
**
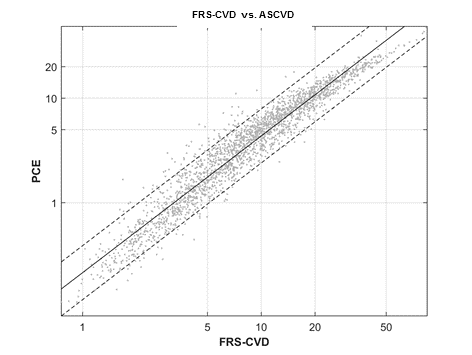
**
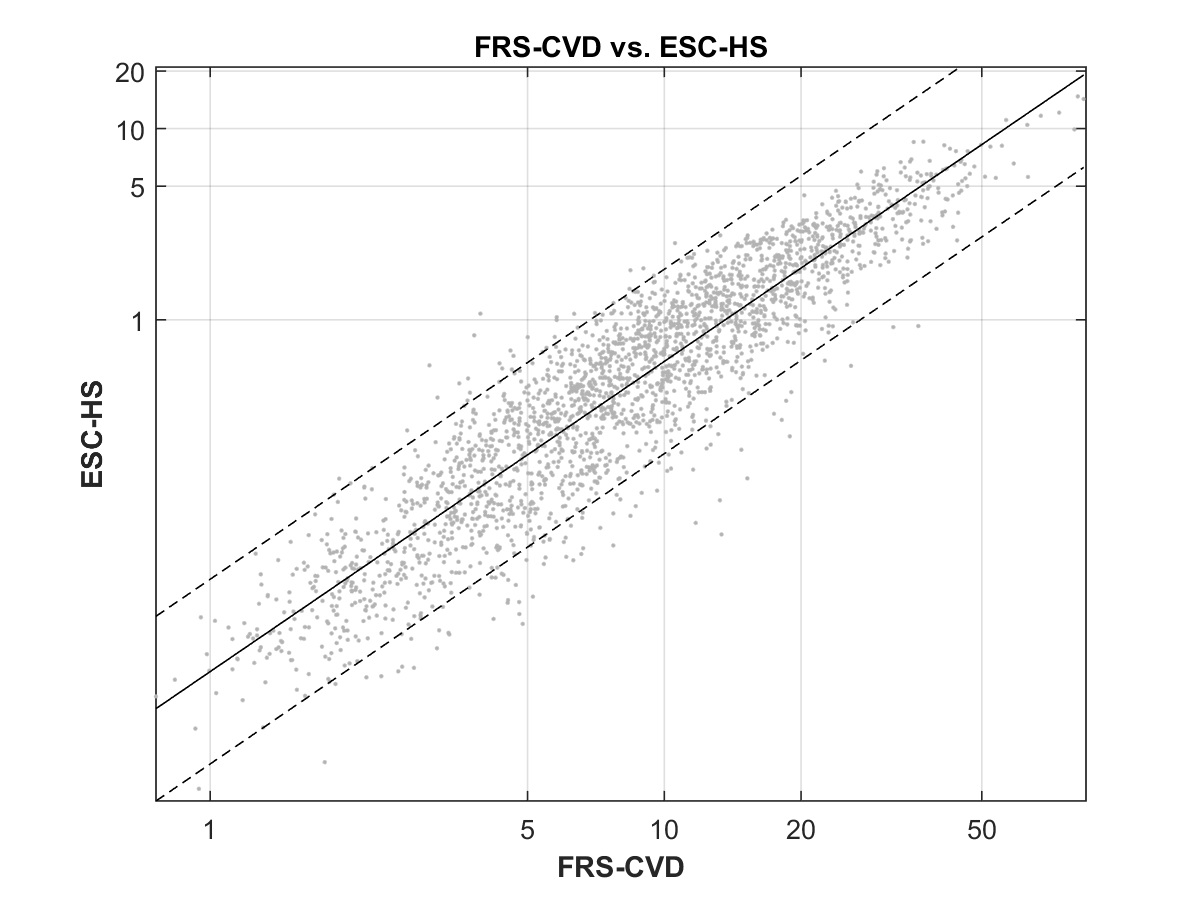


**Supplementary Figure 2**

**
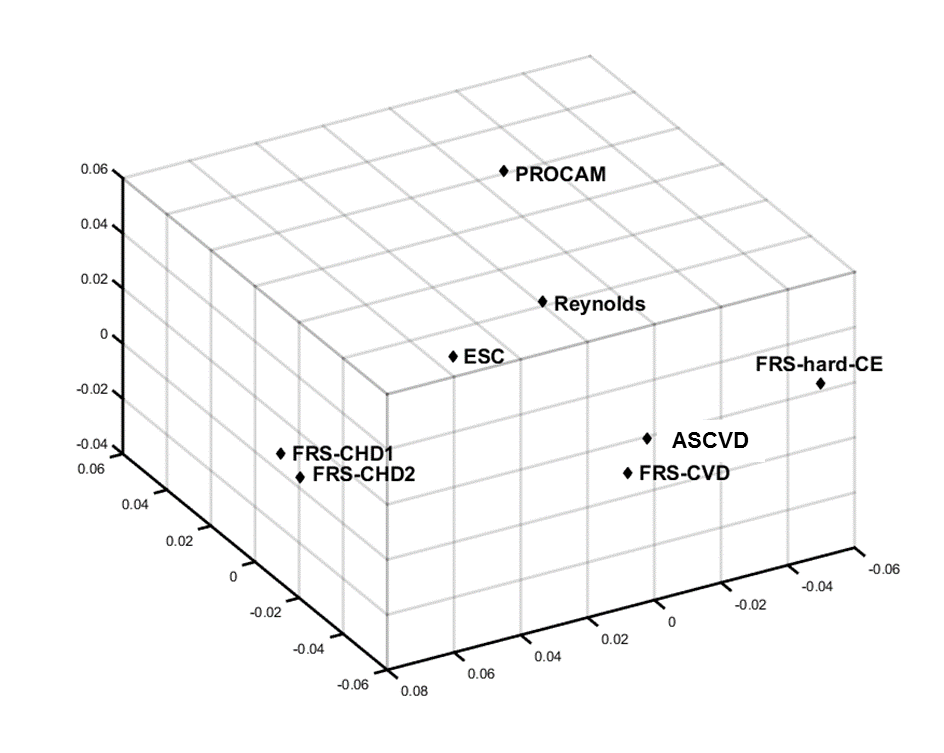
**
